# Triassic hydrothermal chimneys from the Ordos Basin of Northern China

**DOI:** 10.1038/s41598-021-02053-0

**Published:** 2021-11-22

**Authors:** Jiyuan You, Yiqun Liu, Dingwu Zhou, Yiyao Yang

**Affiliations:** 1 School of Energy Engineering, Yulin University, Yulin, 719000 China; 2 State Key Laboratory of Continental Dynamics, Department of Geology, Northwest University, Xi’an, 710069 China

**Keywords:** Solid Earth sciences, Geochemistry, Geology, Mineralogy, Volcanology

## Abstract

Because few well-preserved hydrothermal chimneys have been found in terrestrial sedimentary rocks, research on paleo-thermal vents in geological history is relatively sparse. In this study, we present our original discovery of “hydrothermal chimneys” from the Chang 7 source rocks of the Triassic Yanchang Formation in the Ordos Basin, China, and provide the best evidence for deciphering hydrothermal activity preserved in the geological record (i.e., sedimentary rocks). Three possible chimney samples (i.e., samples 1551.6, 1551.6–2 and 1574.4) were collected for this study; they were interbedded with mudstones and oil shales, indicative of a deep-lake sedimentary environment. All three samples consist mainly of anhydrite, pyrite, and dolomite with the formation of mineral zoning across the walls of these structures, suggesting a sulfate-dominated stage and a carbonate-sulfide replacement stage. Moreover, their in situ geochemistry is characterized by high Eu, U, Th, Sr, Mn and U/Th ratios, which are typical indicators of hydrothermal vents. In addition, their S isotopes range from 7.89% to 10.88%, near the values of magma sulfur, implying a possible magmatic trigger for these hydrothermal vents. All this evidence shows that the Triassic sedimentary rocks of the Ordos Basin probably contain hydrothermal chimneys. Comparing ancient hydrothermal chimneys to modern hydrothermal chimneys, we should note the important implications of ancient chimneys; their formation mechanism may have been related to oil production, and they are possible indicators for future oil investigations. Further, they have great significance for studying the hydrothermal properties of primary dolomite.

## Introduction

A submarine hydrothermal chimney is a tubular structure formed by the eruption of hydrothermal fluid on the seabed. Its structure and chemical composition are closely related to the properties of the hydrothermal fluid from which the chimney body formed. In the wall of a chimney with a thickness of 1 to 3 cm, there are very large geochemical, thermal and mineral combination gradients. A chimney contains rich information about the properties of hydrothermal fluids and mineralization temperatures and records the growth process and growth history of the chimney body^1,2^. Based on the research results of different hydrothermal chimneys, scientists have classified them into 3 categories according to temperature and mineral composition: "black chimney", "white chimney" and "silicon chimney"^3,4^. Over the years, research on the cause and growth mechanism of hydrothermal chimneys has received attention from the international academic community. Reconstructing the formation environment and growth mechanism of the chimney body according to the different structural compositions of the chimney body is one of the important aspects. The development of related research also involves many topics, such as deep sea material circulation, energy transfer, the genesis of mineral deposits and even the origin of life^5,6^. However. The amount of research on hydrothermal chimneys in modern lakes is relatively low, and there is even less research on lacustrine hydrothermal chimneys in geological history. The reason is that although they are important specimens for studying hydrothermal mineralization and activity and extreme environments throughout geological history, ancient hydrothermal chimneys are easily destroyed during the burial process, and it is difficult to find complete samples. Fortunately, abundant samples of hydrothermal chimneys from the Chang 7 section of the Triassic Yanchang Formation in the Ordos Basin were recently discovered by our team. Their tubular shapes and inner and outer walls are fully developed, so they are particularly valuable. They have opened up a new way to study the structural characteristics and growth history of hydrothermal chimneys in continental basins in the Late Triassic. and provide an excellent opportunity for the comparative study of modern and ancient hydrothermal chimneys.

High-quality source rocks have been developed in the Chang 7 Member of the Upper Triassic Yanchang Formation on the southern margin of the Ordos Basin. Previous studies^7,8^ have also confirmed the occurrence of hydrothermal activity during the deposition of the source rocks. Based on research about modern hydrothermal deposition and submarine black chimneys, we study the morphology, material composition, structure, mineral characteristics, geochemical characteristics and formation stages of hydrothermal chimneys, and we compare them with chimney samples from modern ocean floors and continental hydrothermal activity and discuss their geological significance.

## Geological setting

**Figure 1:**
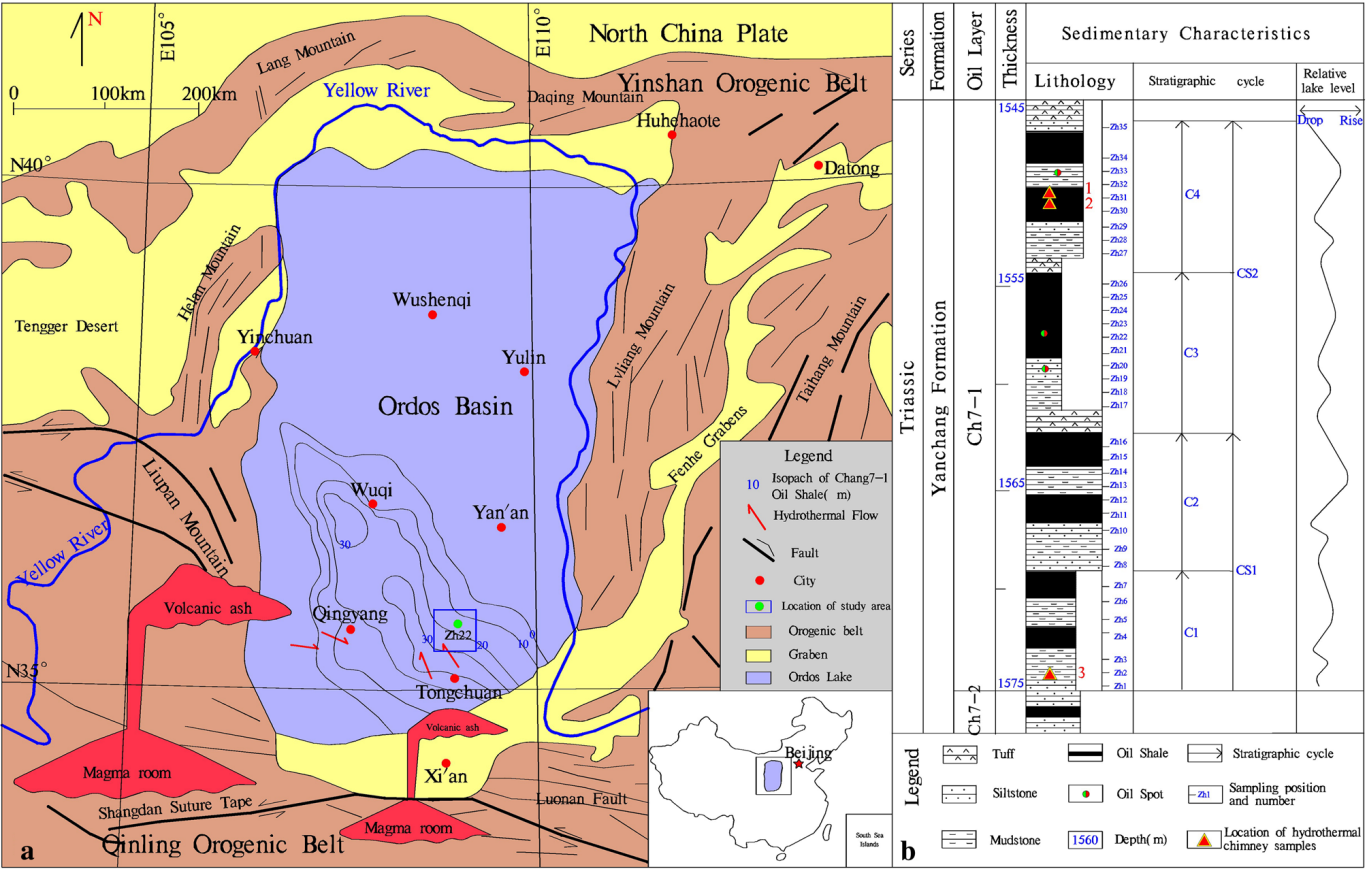
Regional geological map of the Ordos Basin and stratigraphic column with sampling positions (Map modified from reference^8^, and it is drawn with Mapgis6.7, version number is 6.7, the map from URL link: https://www.tandfonline.com/doi/full/10.1080/08120099.2019.1612783).

The Ordos Basin is located in Central China. It is a lacustrine sedimentary basin that formed during the Mesozoic. The basin area is approximately 25 × 10^4^ km^2^^9^. Its periphery is surrounded by Cenozoic fault basins. The western part of the basin is a tectonic thrust belt and the Liupan Mountains, and the southern part is the Weihe Basin and the Weibei uplift. The Jinxi flexural belt is located in the eastern part of the basin. The northern part of the basin is adjacent to the Yimeng uplift and the Hetao Basin uplift. The overall contours of the basin form a roughly north–south rectangle (Fig. 1). In the Late Triassic, the basin contained the largest area of lake water, which covered almost the entire basin. Deep lake facies developed on the southern margin of the basin, and thick layers of dark mudstone and oil shale that compose the Chang 7 member of the Yanchang Formation were deposited, with a thickness of up to 100 m. This set of high-quality source rocks has strong hydrocarbon generation and expulsion capabilities and is the main Mesozoic source rock in the basin. According to sedimentary cycles and lithologic combination characteristics, it can be divided into 10 oil layer groups (Chang 10-Chang l) from bottom to top. The Chang 7 section is subdivided into 3 subsections: Chang 7–3, Chang 7–2 and Chang 7–1. During the Chang 7 sedimentation period, the basin subsided, and the depth of the lake increased, reaching a depth of 60–80 m in the southern part of the basin. The area of the lake basin increased, and a large area of deep water appeared, covering almost the entire Ordos Basin^8^. In the context of strong tectonic activity in this region, basal ruptures may have been activated^8^, which created conditions for the development of hydrothermal chimneys in the basin.

## Geological characteristics of hydrothermal chimneys

**Figure 2:**
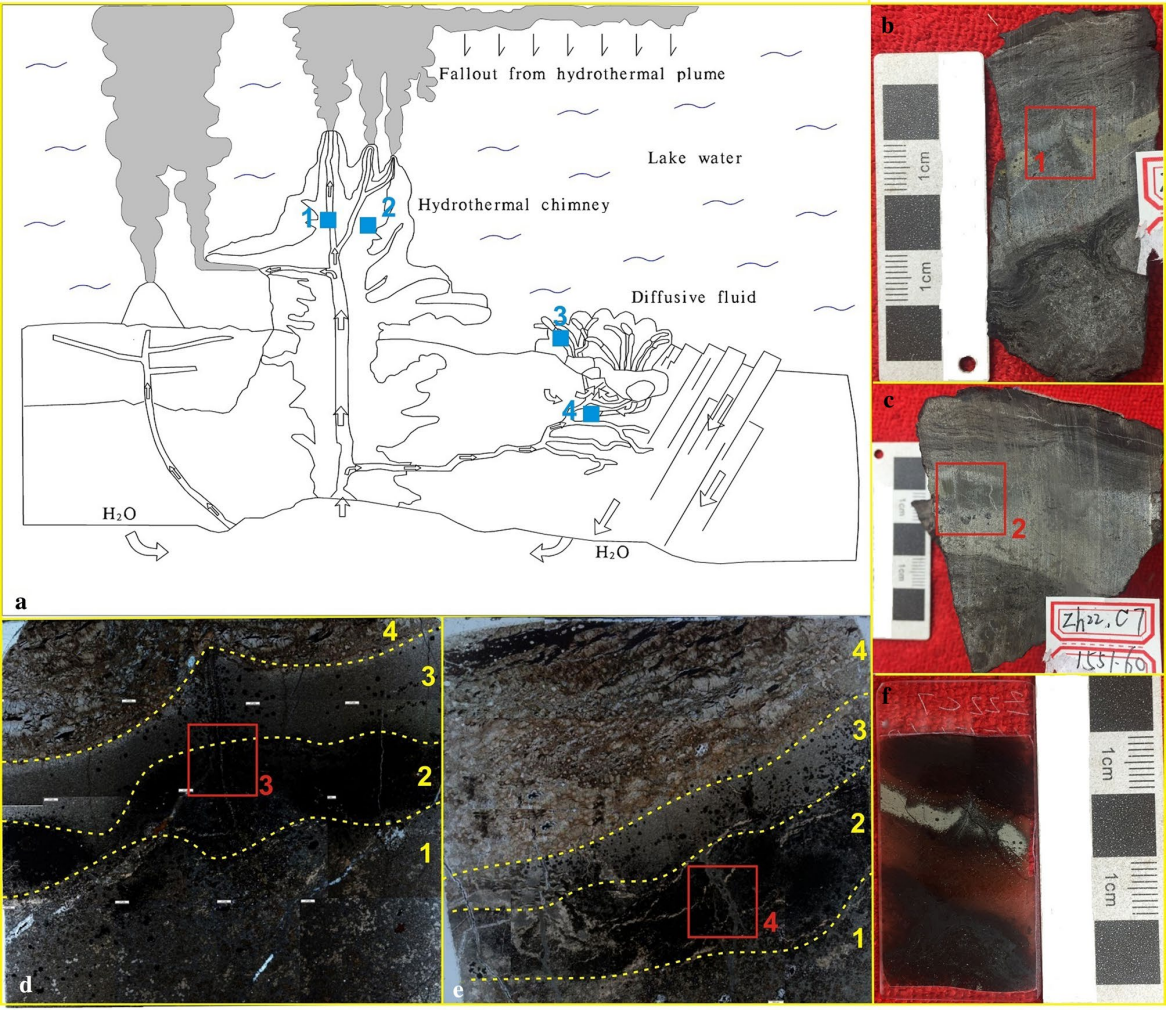
Characteristics of hydrothermal chimneys in the study area (All photos in Fig. 2 were taken by You Jiyuan). (**a**) Sketch of typical hydrothermal chimney (Map modified from reference^1^, (The Figure is drawn with Mapgis 6.7, version number is 6.7, the map from URL link: https://www.tandfonline.com/doi/full/10.1080/08120099.2019.1612783). (**b**) Hydrothermal chimney, 6 cm × 4 cm, sample No. 1551.6, the sample was taken from the blue box No. 1 in (**a**); (**c**) Hydrothermal chimney, 7 cm × 6 cm, sample No. 1551.6–2, the sample was taken from the blue box No. 4 in (**a**); (**d**) Enlarged view of the No. 1 red frame in panel b, in which layer 1 is dolomite with a radial structure, layer 2 is pyrite, layer 3 is a dolomite + calcite layer, and layer 4 is calcite + dolomite + anhydrite + animal microfossils. 2.5X magnification, with cross-polarized light. (**e**) Enlarged view of the No. 2 red frame in panel (**c**), in which layer 1 is calcite + dolomite + anhydrite with a radial structure, layer 2 is pyrite, layer 3 is a dolomite + anhydrite layer, layer 4 is calcite + dolomite + anhydrite + animal microfossils. at 2.5X magnification, with cross-polarized light. (**f**) Thin slices of hydrothermal channel samples.

The hydrothermal chimneys studied in this paper were found in the Chang 7–1 section of the Triassic Yanchang Formation in the Z22 well, which is located in the southern part of the Ordos Basin. The strata are dominated by dark siltstones and mudstones, with iron dolomite, carbonates, and thin siliceous rocks. Gravity flows such as sandy debris flows and turbidites are observed, and abundant fossils of animal microorganisms and plant debris are also distributed. In this paper, three typical hydrothermal chimneys in the Zh22 well were selected for detailed analysis (Fig. 2b,c,f). Numerous polished thin sections from the three chimney samples (1551.6, 1551.6–2 and 1574.4) were examined microscopically to determine the mineral contents and textural characteristics of the hydrothermal chimney walls. Analyses of the same sections were made with an electron microprobe to accurately determine specific mineral compositions and to document compositional zoning within individual grains and across chimney walls from interior to exterior.

**Figure 3:**
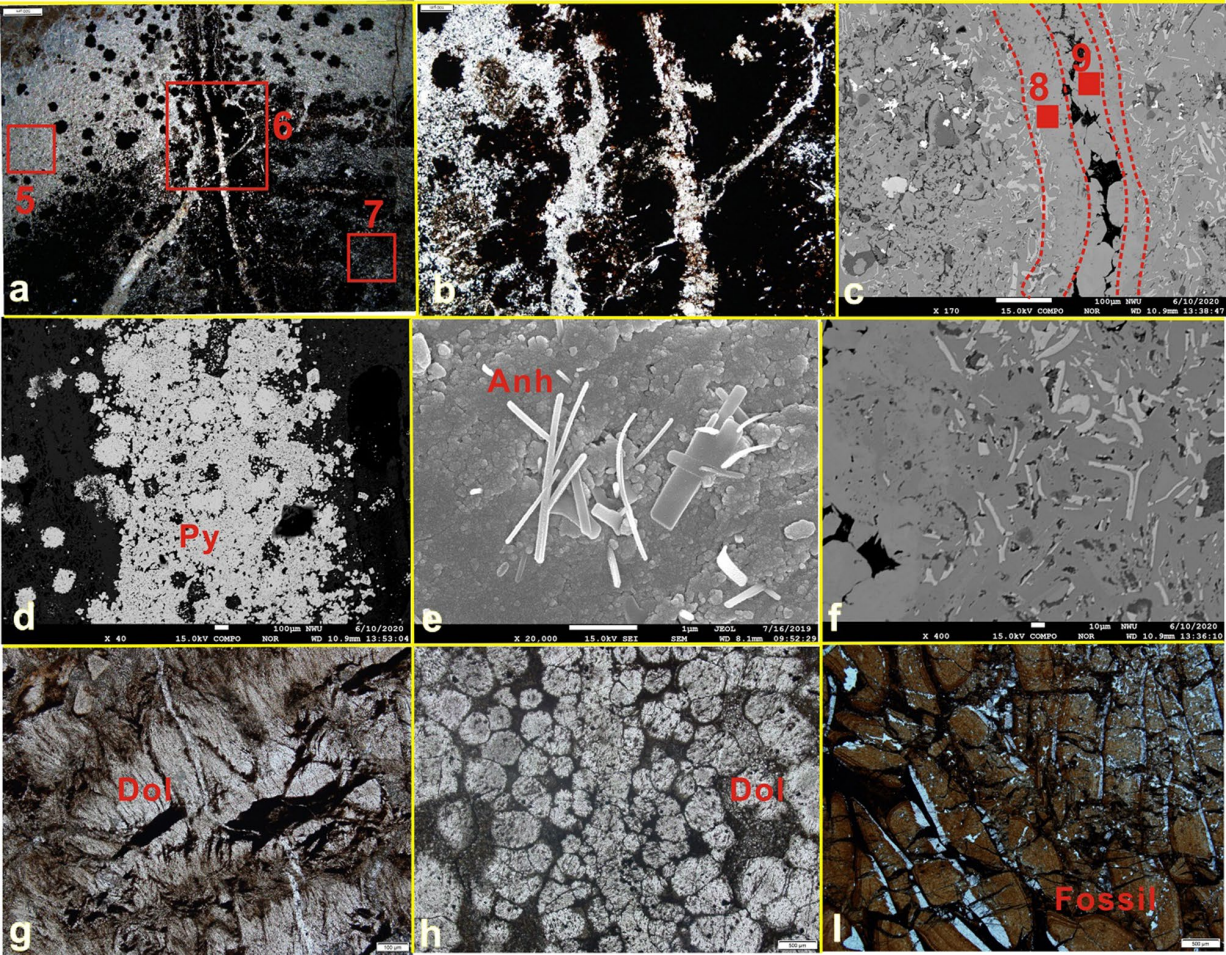
Microscopic photo of hydrothermal chimney. (**a**) Enlarged view of the No. 3 red frame in Fig. 2d, hydrothermal channel, 2.5X, cross-polarized light. (**b**) Enlarged view of the No. 6 red frame in (**a**), hydrothermal channel, 10X, ross-polarized light. (**c**) Enlarged view of the No. 6 red frame in (**a**), hydrothermal channel, BSE. (**d**) Enlarged view of the No. 9 red frame in (**c**), pyrite, BSE. (**e**) Enlarged view of the No. 8 red frame in Fig. 8, anhydrite with long strip, SEM. (**f**) The bone-like structure in (**c**), BSE. (**g**) Enlarged view of the No. 5 red frame in (**a**), the radial structure of calcite, 2.5X, cross-polarized light. (**h**) Enlarged view of the No. 2 red frame in Fig. 2f shows that the breccia dolomite develops a ring zone, 2.5X, cross-polarized light. (**i**) Microfauna fossils deposited in No. 3 red frame in Fig. 2f.

① The cores from 1549 to 1555 m are dominated by black oil shale, and dolomite and siliceous rocks are locally developed (Fig. 2a). Sandstone dikes cut through the oil shale along the fault (Fig. 2b). The dolomite fluid and the surrounding rock show a co-sedimentary relationship (Fig. 2c). There are also obvious hydrothermal channels. The above characteristics indicate that the tectonic activity in the study area is relatively strong and may be located in a deep fault zone. ② Hydrothermal chimneys are developed in oil shale and have a columnar shape when viewed in cross section, with many branches converging upward (Figs. 2f, 3a–c). The results of mineralogical analysis show that the minerals in the hydrothermal chimneys are mainly pyrite, anhydrite, barite, calcite, and dolomite and small amounts of quartz and albite. The outermost skin of black smokers bears fine-grained pyrrhotite (Fe_1-x_S), pyrite (FeS_2_) (Fig. 3d) and dolomite (CaMg(CO_3_)_2_) with textures matching those in mineralogically similar black plume particulate assemblages. Within chimney walls, a pyrrhotite-free sulfide and carbonate assemblage of coarser, more euhedral FeS2 (pyrite and/or marcasite), dolomites and calcites is present. These sulfides and carbonates often replace anhydrite outward along crystallographic planes in the sulfate (Fig. 3e). ③ Observations of the vertical cross section of the hydrothermal chimney channel show that the channel structure of the chimney is clearly visible. The chimney is composed of a channel, inner wall, outer wall and surrounding rock. The main channel is associated with branch channels, and the minerals and structures of the channel are zoned (Fig. 2d,e). ④ The parallel cross section of the hydrothermal chimney channel contains 4 sedimentary layers. The bottom of the hydrothermal chimney is composed of dolomite and calcite and develops a chicken bone-like structure (layer 1 in Fig. 2d, layer 1 in Figs. 2e, 3f); Pyrite layers are distributed on both sides of the hydrothermal chimney (layer 2 in Fig. 2d, layer 2 in Fig. 2e) and are formed by the precipitation of breccia pyrite supplied by the hydrothermal chimney (Fig. 3a). Dolomite and calcite with breccia structure develop in the middle layer of the hydrothermal chimney (layer 3 in Fig. 2d, layer 3 in Fig. 2e); dolomite and calcite have a good degree of crystallinity, and the grains develop zoning (Fig. 3h), which is explained as the natural growth of carbonate minerals in the hydrothermal environment. The breccia structure, saddle-like structure, radial structure, etc., often appear at the top of the hydrothermal chimney (layer 4 in Fig. 2d, layer 4 in Fig. 2e), which is composed of dolomite, calcite, pyrite and microfauna fossils (Fig. 3g,i). The above characteristics confirm the existence of hydrothermal chimneys, and they record the development process of the chimneys.

## Sample analysis and experimental methods

### sample collection and processing

From the bottom layer to the top layer, the researcher collected 11 samples of mudstone, 4 samples of argillaceous siltstone, and 20 samples of hydrothermal sedimentary rock from the Chang 7–1 section of the Yanchang Formation of the Triassic in the Zh22 well, with a total of 35 samples, in which hydrothermal chimneys were found in samples 1551.6, 15,516.-2 and 1574.4. During the sampling process, the influence of the mechanical differentiation on sediment composition and the tendency of sampling differences are minimized or eliminated. The sample collection and processing are described: ① Three samples about hydrothermal chimney were ground into ordinary rock flakes. After observing the sedimentary structure of the rock specimen and the composition, microstructure and fluorescence characteristics of the mineral under the microscope, the samples with complete morphology were selected to be made into electron probe sheets and SEM samples to determine the main elements, mineral components and microscopic mineral structure. ② The geochemical composition of pyrite particles, coarse-grained dolomite and coarse-grained calcite was further analysed with LA-ICP-MS. Since the laser beam spot of this method is 36 μm, which is much larger than that of micro-crystalline minerals and secondary enlarged mineral particles, only coarse-grained mineral particles can be analysed. ③ For the preparation of SEM samples, according to the needs of the experiment, a cube of 0.5 × 0.5 × 0.5 cm was made. The surface structure of the 6 sections was intact and was not damaged. It was used after being sputtered with gold for conductive treatment. After completing the above work, the rock samples were selected according to the characteristics of the samples and the research needs for the main and trace elements, scanning electron microscopy and other tests.

### Analysis method

1. A total of 48 sets of data were completed for in situ trace element and rare earth element experiments, which were completed at the State Key Laboratory of Continental Dynamics, Northwest University. using an Agilent 7500 ICP-MS instrument attached to a 193 nm excimer laser ablation system (Geolas 2005). All measurements were performed in time-resolved mode with helium as the carrier gas. The carrier flow was optimized to obtain the maximum signal intensity for ^238^U + , while keeping the ThO^+^/Th^+^ ratio below 0.5% and the U^+^/Th^+^ ratio close to 1 for the reference glass (SRM 610, National Institute of Standards and Technology)^10^.
2. The main element was tested using a JXA-8100 electron probe system (15 kv, beam 1 × 10^-8^A, beam spot 1-5 μm) and an X-ray energy spectrometer (LINK ISIS300, UK) to analyse the main elemental constituents and contents of the different compositions of hydrothermal minerals, such as iron dolomite and pyrite.
3. The mineral characteristics and structure of the rock samples were observed using SEM (Model S-3200, Hitachi, Ltd.) and an environmental SEM (Quanta Model 400, Phillips). The observation range is approximately 1 cm^2^. A larger range of progressive scans was performed on the target sample (we can see the hydrothermal chimney) and the control sample (no hydrothermal chimney sample).
4. Sulphur isotope analysis of pyrite was completed at the State Key Laboratory of Continental Dynamics, Northwest University. The laser ablation system was manufactured by Resso, model RESOlution S-155. The ArF excimer laser generator produces a 193 nm deep ultraviolet beam that is focused on the sulphide surface by a homogenized optical path. The diameter of the laser beam spot is generally 33 μm, the ablation frequency is 10 Hz, and the ablation is performed for 40 s. The high-purity helium gas is used as a carrier gas and mixed with argon gas and nitrogen gas to enter the mass spectrometer. The multi-receiver plasma mass spectrometer was manufactured by the Nu Instrument Corporation using the model Nu Plasma II. The ^34^S/^32^S ratio of the standard sample and the sample point was obtained by direct test, and the δ^34^SCDT value was calculated by the external standard correction (SSB method). The standards used were the international sulphide standard NBS-123 sphalerite and the laboratory internal standard WS-1 pyrite (Fig. 5e).

## Results

The experimental analysis of electron probe, major elements, trace elements, rare earth elements and in-situ S isotope of the hydrothermal chimney samples in the study area has been completed. The specific experimental results are as follows:

### Electron probe analysis

**Figure 4:**
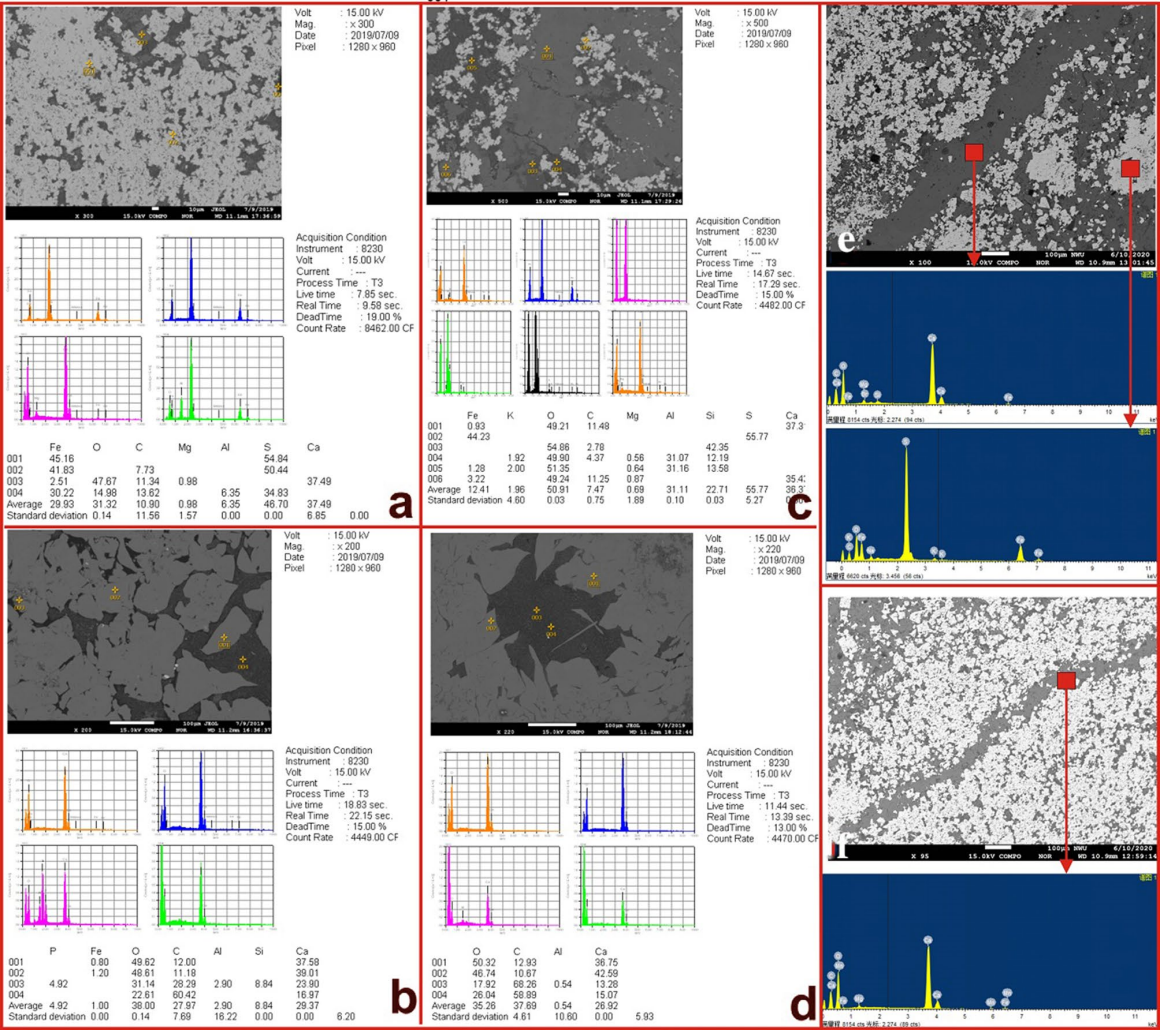
Images of SEM and EDS data for minerals in the study area. (**a**) EDS data for Fig. 2i, white matter is pyrite. (**b**) EDS data for Fig. 2l, calcite. (**c**) EDS data for Fig. 2g, pyrite + iron dolomite + quartz. (**d**) EDS data for Fig. 2l, calcite. Panels (**e**,**f**) show that the hydrothermal channel is composed of dolomite and pyrite.

The experiment results of electronic probe of the hydrothermal chimney samples are shown in Table 1. A total of 19 points of the 3 samples were analyzed. The samples are mainly composed of dolomite, calcite, pyrite, siderite, and feldspar (Fig. 4). In the 1551.6, 1574.4 and 1551.6–2 samples, SiO_2_, Al_2_O_3_, FeO and CaO are the four most abundant elements. In this study, 19 positions of the hydrothermal channel were selected to complete the electron probe test. The results are shown in the Table 1. The experimental points of sample 1551.6 correspond to the 9 yellow stars in Fig. 2a. The SiO_2_ contents of points 7 and 8 were 57.255% and 58.108%, respectively, and the remaining positions contained almost no SiO_2_. The Al_2_O_3_ content of points 7 and 8 were 34.92% and 33.91%, respectively, and the remaining positions had very small contents of Al_2_O_3_, ranging from 0.003% to 0.09%. The FeO contents of points 2 and 4 were 48.754% and 45.756%, respectively, and the remaining positions had very small contents of Al_2_O_3_, ranging from 0.86% to 3.298%. The CaO contents at points 1, 5, 6 and 9 were 55.696%, 55.355%, 60.486% and 58.097%, respectively, and the CaO content in the remaining positions was small. Other major elements, such as MgO, Na_2_O, K_2_O, MnO, TiO_2_ and P_2_O_5_, were rare. Except for point 3, the content of SrO was 0.052% to 0.846%, and the average content was 0.412. In the first four positions of sample 1551.6–2 (The experimental points correspond to the 5 yellow stars in Fig. 2b), CaO was the main element, and the value was located at 52.79 ~ 54.824%; the fifth position was pyrite, and the main elements were Fe and S. In sample 1574.4, a total of 5 dolomite single mineral experiments were completed. Here, CaO was the main element, and the values were located between 37.688 and 44.375.

**Table 1:** Major elements. The experimental points of sample 1551.6.

sample	Experimental point	Lithology	Al2O3	SiO2	CO2	MgO	SrO	FeO	Total
1551.6	1	Calcite	0	0	42.53	0.019	0.631	1.075	100
1551.6	2	Siderite	0.05	0.027	38.11	2.932	0.067	48.754	100
1551.6	3	Pyrite	0	0	0	0	0	48.145	48.145
1551.6	4	Siderite	0.08	0	37.48	2.584	0.052	45.756	100
1551.6	5	Calcite	0.01	0	40.46	0.397	0.12	3.298	100
1551.6	6	Calcite	0.08	0	37.42	0.224	0.601	0.86	100
1551.6	7	Feldspar	34.9	57.26		1.707	0.303	1.217	100
1551.6	8	Feldspar	33.9	58.11		1.95	0.311	1.272	100
1551.6	9	Calcite	0.09	0	39.45	0.265	0.846	0.962	100
1551.6–2	1	Calcite	0.02	0	42.02	0.02	0.65	1.05	100
1551.6–2	2	Calcite	0.01	0	40.98	0.018	0.624	1.87	100
1551.6–2	3	Calcite	0.08	0	41.57	0.021	0.694	2.11	100
1551.6–2	4	Calcite	0.24	0	41.23	0.01	0.776	2.05	100
1551.6–2	5	Pyrite	0	0	0	0	0	49.045	49.045
1574.4	1	Calcite	0.05	0	37.69	0.447	0.108	0.399	100
1574.4	2	Calcite	0.14	0	39.42	0.766	0.002	0.625	100
1574.4	3	dolomite	0	0	43.9	8.954	0.134	17.147	100
1574.4	4	dolomite	0.02	1.449	43.78	9.339	0.183	12.847	100
1574.4	5	dolomite	0.24	0	44.38	10.558	0.147	13.207	100

### Trace elements

**Figure 5:**
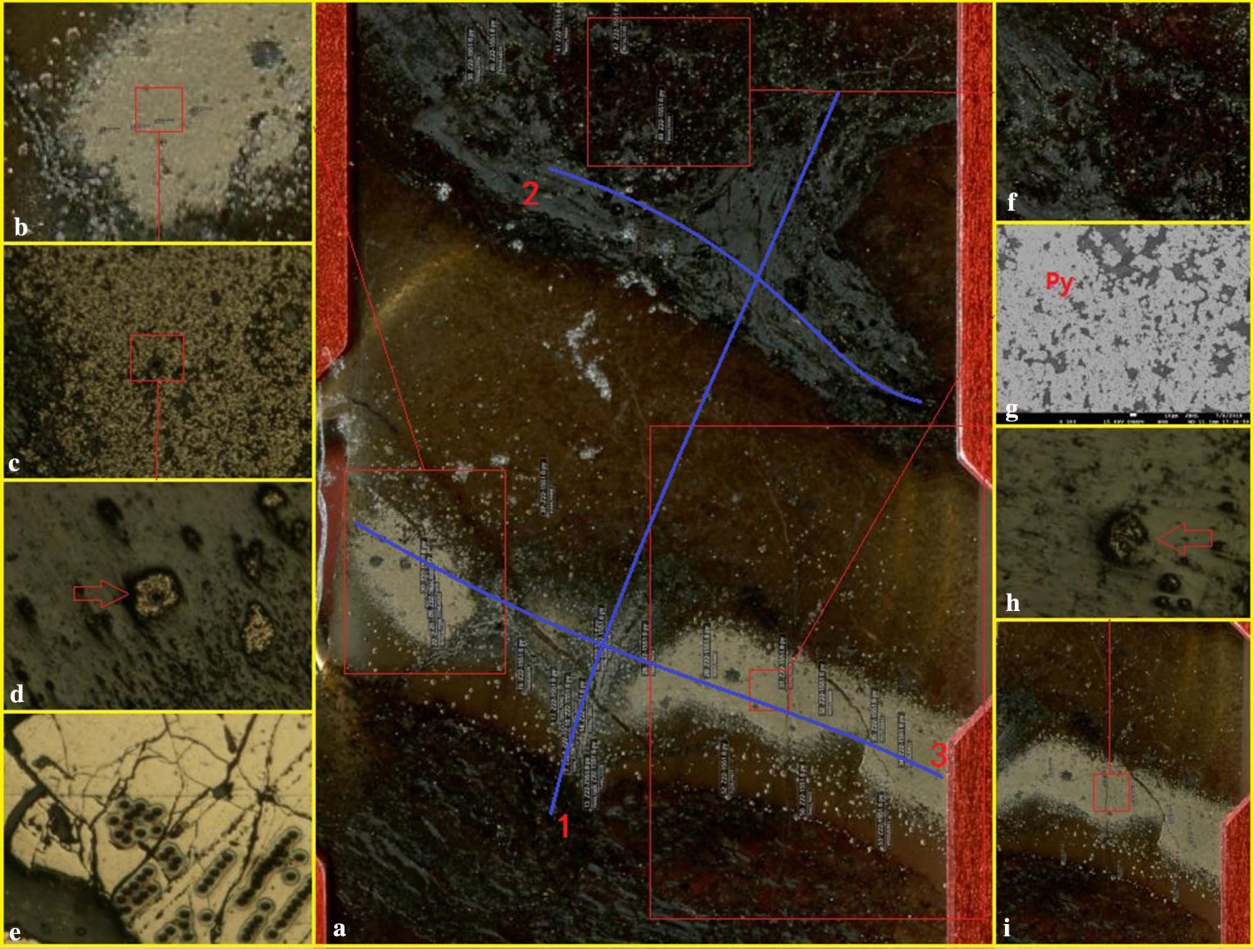
Analytical points for in situ trace elements and sulfur isotopes around pyrite in the hydrothermal chimney. (**a**) Macroscopic photograph of the hydrothermal vent and experimental point, where the three blue lines indicate the positions of the in situ trace elements. (**b**) Partially enlarged photograph of panel (**a**). (**c**) Partially enlarged photograph of panel (**b**). (**d**) Partially enlarged photograph of panel (**c**). (**e**) Standard sample of pyrite from the State Key Laboratory of Continental Dynamics, Northwest University. (**f**) Partially enlarged photo of the red frame in panel (**a**). (**g**) Backscattered election image of panel (**a**) at 130X magnification. (**h**) Partially enlarged photograph of panel (**i**). (**i**) Partially enlarged photograph in the red box at the right of panel (**a**).

**Figure 6:**
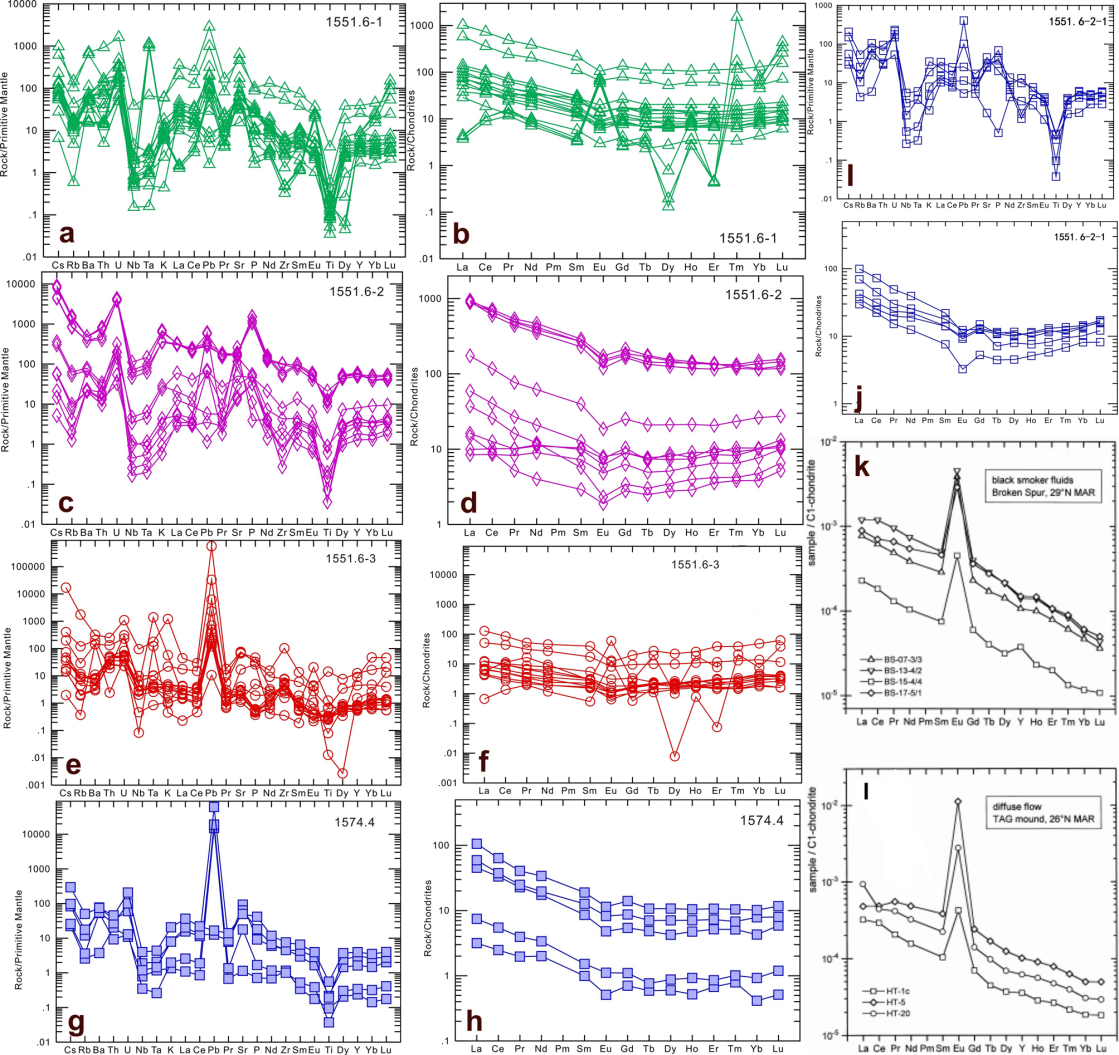
Standardized spider diagrams of REEs/chondrites and trace elements/primitive mantle from the hydrothermal chimney (Note: The data of panels (**a**,**b**) are from blue line No. 1 in Fig. 5, hydrothermal dolomite; the data of panels (**c**,**d**) are from blue line No. 2 in Fig. 5, Mixture of hydrothermal sedimentary rock and mudstone; and the data of panels (**e**,**f**) are from blue line No. 3 in Fig. 5, hydrothermal pyrite. The data for panels (**g**,**h**) are from sample 1574.4, hydrothermal dolomite. The data for panels (**i**,**j**) are from sample 1551.6-2, hydrothermal dolomite). REYCN patterns of (**k**) high-temperature hydrothermal fluids from the Broken Spur vent site, 29° N Mid-Atlantic Ridge, and (**l**) low-temperature diffuse hydrothermal flow at the TAG hydrothermal mound, 26° N Mid-Atlantic Ridge^27^.

In this study, three typical samples about hydrothermal chimney were selected, and a total of 49 positions were used for in situ trace elemental analysis. The results are shown in Table 2. From the standard spider web map of trace elements (Fig. 6a,c,e,g,i) shows that except for U, Nb, Pb, Ti and other elements, the content fluctuates greatly, and the other elements have more consistent trends. ① The in-situ trace element experiment of sample 1551.6 was completed around three blue lines (the blue lines 123 in Fig. 5 represent different positions of the hydrothermal chimney respectively). Line 1 represents the passage of the hydrothermal chimney. Studies have shown that: the U, Pb, and Sr contents are significantly positive, indicating that the material source of the jet channel is complex. Mixed with shell source material; Some points of Eu are significantly positive, and some are weak positive, which explains that the spray channel mineral is formed in a high temperature environment, and the temperature away from the nozzle is lowered. Line 2 represents the top position away from the hydrothermal chimney, corresponding to layer 4 in Fig. 2d. Studies have shown that: U and P are significantly abnormal, indicating that the hydrothermal fluid brings nutrients to promote the improvement of the ancient productivity. Line 3 represents the pyrite layer of the hydrothermal chimney, corresponding to layer 3 in Fig. 2d. where Pb is significantly abnormal, close to 20,000, and Eu shows a weak positive anomaly. ② The in situ trace elemental analysis of sample 1574.4 showed that U and Pb were significantly positive and that Eu shows a weak negative anomaly. ③ The in situ trace elemental analysis of sample 1551.6–2-1 showed that U and Pb were significantly positive and that Eu showed a weak negative anomaly. According to REYCN patterns of (Fig. 6k) high-temperature hydrothermal fluids from the Broken Spur vent site, 29°N Mid-Atlantic Ridge, and (Fig. 6L) low-temperature diffuse hydrothermal flow at the TAG hydrothermal mound, 26°N Mid-Atlantic Ridge. They all show that Eu is abnormal. In Fig. 6b,f of this study, some samples also show positive abnormalities of Eu.

**Table 2:** Trace elements. We completed a total of 39 experiments on sample 1551.6, 5 experiments on sample 1551.6–2, and 5 experiments on sample 1574.4.

Contents (ESIS)		Na	Al	Si	P	K	Sc	Ti	V	Cr	Mn	Fe	Co
Point NO.	Sample NO.	ppm	ppm	ppm	ppm	ppm	ppm	ppm	ppm	ppm	ppm	ppm	ppm
191029A004	1551.6	963.14	17581.44	38169.46	2471.92	4520.26	12.56	545.06	53.12	12.49	6275.65	25676.90	3.08
191029A005	1551.6	369.39	7437.74	15450.90	990.61	1629.28	6.26	161.42	22.43	5.29	5764.14	21586.31	0.41
191029A006	1551.6	641.66	3186.71	7016.98	2912.07	586.65	3.47	115.28	30.08	3.39	1381.46	20171.38	0.36
191029A007	1551.6	184.53	1047.44	2640.56	721.37	109.58	1.13	67.37	11.50	1.83	3768.78	15834.76	0.20
191029A008	1551.6	370.05	10852.47	18076.07	1068.33	1786.36	4.40	280.68	22.71	2.54	6150.66	33524.98	0.60
191029A009	1551.6	3935.04	90791.60	154183.30	9715.28	14807.70	49.49	5339.96	263.44	33.95	70005.62	69.63	9.94
191029A010	1551.6	2538.11	88427.62	136830.32	8231.99	15160.96	18.76	806.36	185.03	35.13	38995.88	69.63	5.37
191029A011	1551.6	491.22	11508.08	18452.82	2339.17	2079.35	4.22	120.22	30.83	4.88	3455.17	56862.45	1.35
191029A012	1551.6	414.68	8029.74	12879.35	3543.06	1459.31	4.81	45.39	27.52	3.34	4591.53	54979.52	1.34
191029A013	1551.6	363.79	11118.65	18703.33	2572.67	1984.76	5.77	133.39	34.22	4.63	4781.01	47449.54	1.05
191029A017	1551.6	563.13	15286.86	45501.78	932.14	2563.29	2.19	102.13	18.08	2.28	2337.31	16975.77	0.32
191029A018	1551.6	677.44	19907.03	32011.91	3063.88	3349.08	4.07	349.87	19.66	1.97	3607.08	21224.82	0.17
191029A019	1551.6	540.55	16910.22	27230.67	3069.21	2860.60	4.31	187.47	18.85	1.78	3630.04	22216.01	0.10
191029A020	1551.6	31172.09	313075.10	773565.74	141460.32	91921.53	64.72	11576.79	1107.18	272.26	13343.10	351465.55	26.22
191029A021	1551.6	32833.63	294468.59	736444.41	145607.61	88142.93	67.95	14588.87	1022.30	260.96	13704.09	382389.50	26.74
191029A022	1551.6	35522.38	505871.77	1214263.84	114314.13	155972.85	98.67	16397.36	1887.82	466.01	23553.87	606258.07	28.96
191029A023	1551.6	38365.14	544412.09	1291868.56	99907.48	172095.31	102.15	27440.39	2009.82	519.17	23825.97	642534.96	49.55
191029A024	1551.6	645.98	1789.41	5532.23	5042.71	467.38	7.94	94.95	53.99	4.91	2273.82	38646.14	3.39
191029A025	1551.6	440.60	471.94	1539.89	3045.32	144.09	1.31	46.72	12.45	1.70	477.20	4932.30	0.18
191029A026	1551.6	506.97	897.80	2613.52	3086.83	230.63	1.55	46.95	13.79	1.99	523.92	5883.04	0.20
191029A030	1551.6	473.35	4049.45	12372.71	1050.96	1085.12	4.09	198.28	31.11	5.07	6344.23	44434.11	7.73
191029A031	1551.6	422.58	3552.88	18675.73	370.78	801.78	4.30	254.78	14.81	4.48	3565.63	10281.71	0.49
191029A032	1551.6	2353.27	26985.12	81726.98	5203.03	7149.68	14.96	1154.74	124.06	25.88	4756.49	55962.99	5.52
191029A033	1551.6	1569.91	23318.04	52466.51	4439.21	6000.68	15.47	805.40	88.87	21.46	3882.01	36563.25	10.35
191029A034	1551.6	193.60	8419.88	11524.00	53.02	1213.40	1.03	398.29	9.98	1.00	281.38	71.09	0.13
191029A035	1551.6	48.71	1979.05	2875.46	474.86	277.74	0.42	102.63	3.70	0.84	163.08	71.09	23.20
191029A036	1551.6	20570.92	1560523.00	2459778.14	3041.48	301399.64	36.96	18668.89	1366.29	167.24	11767.81	24948848.87	1691.05
191029A037	1551.6	1968.66	28380.87	41875.31	3094.80	5483.94	4.32	884.47	48.74	9.35	3527.10	3508867.87	50.35
191029A038	1551.6	47.15	4152.74	5265.87	44.13	469.82	1.07	321.84	4.19	1.18	184.29	71.09	0.23
191029A039	1551.6	143.74	7406.93	9902.13	57.03	971.55	1.12	819.25	6.92	1.42	229.75	71.09	0.17
191029A043	1551.6	44.83	3038.22	3888.44	35.57	246.18	0.70	449.69	2.51	0.31	141.90	71.09	0.05
191029A044	1551.6	74.57	6348.83	8584.52	42.87	812.50	0.90	406.46	4.54	0.63	172.72	71.09	0.07
191029A045	1551.6	81.57	7583.10	10272.80	48.59	1038.41	0.94	360.12	5.55	0.82	227.66	71.09	0.10
191029A046	1551.6	637.71	7144.37	11534.66	180.84	1039.02	0.52	333.03	5.67	0.98	315.06	71.09	36.07
191029A047	1551.6	118.54	2239.33	3570.45	67.71	340.26	0.18	122.17	2.40	0.68	143.98	71.09	52.06
191029A048	1551.6	248.84	2830.62	4264.25	159.51	493.27	0.23	48.26	3.14	0.89	393.56	71.09	28.82
191029A049	1551.6	1015.02	11644.97	18604.76	867.47	2013.18	1.19	281.02	10.10	2.30	4001.78	1401236.30	82.47
191029A050	1551.6	403.80	7562.46	17624.79	1715.73	1982.57	6.32	250.56	33.22	8.87	5918.08	29963.46	0.44
191029A051	1551.6	1007.93	18762.32	40918.43	3946.80	5132.06	8.73	722.40	67.69	17.84	5770.54	34189.27	1.08
191029A052	1551.6-2	148429.27	25225.25	496334.67	1914.06	8834.71	10.08	618.51	100.50	19.67	5555.42	30738.47	1.33
191029A056	1551.6-2	12151.23	8778.86	49837.63	6545.19	1978.28	7.36	48.70	50.76	4.65	4810.00	42017.98	0.32
191029A057	1551.6-2	658.56	4076.90	9966.83	4042.36	1158.37	5.77	124.65	36.03	5.50	1783.94	21932.58	0.45
191029A058	1551.6-2	993.09	33284.31	51858.96	3609.95	4738.96	3.88	578.72	14.59	2.28	3095.20	27037.39	0.19
191029A059	1551.6-2	64.85	4903.88	6434.63	48.05	481.78	1.05	441.10	6.41	1.36	207.81	71.09	0.13
191029B012	1574.4	271.28	592.28	1543.45	2516.77	113.35	2.13	16.58	2.92	1.64	210.33	3089.07	0.18
191029B013	1574.4	2411.93	66892.56	2318040.46	4414.41	14375.04	96.10	1618.07	107.35	69.77	10194.34	442869.50	855.45
191029B017	1574.4	541.43	6891.11	17313.63	152.01	1633.02	17.46	232.91	34.53	8.65	5615.27	87403.35	1.40
191029B018	1574.4	1116.53	7331.88	23169.35	213.58	1871.51	23.12	322.20	37.60	9.48	6000.14	98721.17	9.17
191029B019	1574.4	759.8144848	6588.226017	19794.92617	471.6682226	1584.87534	27.40246095	302.6913236	35.57103662	8.3491601	5777.565781	91545.58875	1.14427743

Contents (ESIS)		Ni	Cu	Zn	Ga	Ge	Rb	Sr	Y	Zr	Nb	Mo	Cs
Point NO.	Sample NO.	ppm	ppm	ppm	ppm	ppm	ppm	ppm	ppm	ppm	ppm	ppm	ppm
191029A004	1551.6	4.32	3.74	24.91	5.04	0.35	27.07	1368.10	26.38	64.26	1.60	1.75	1.79
191029A005	1551.6	1.26	0.30	5.05	1.61	0.33	9.28	2661.81	14.07	30.32	0.62	0.97	0.54
191029A006	1551.6	1.13	2.73	2.83	0.74	0.39	3.01	325.51	41.27	27.82	0.58	1.81	0.22
191029A007	1551.6	0.09	0.00	2.90	0.30	0.00	0.37	782.17	12.95	58.04	0.32	0.21	0.05
191029A008	1551.6	1.86	2.15	5.78	1.57	0.58	6.48	831.80	22.70	76.25	1.13	28.35	0.61
191029A009	1551.6	72.55	43.83	196.57	18.75	5.40	65.61	9711.58	178.73	1096.56	27.19	746.76	7.67
191029A010	1551.6	35.34	35.98	45.87	15.50	7.76	94.79	13480.06	116.51	591.67	3.22	396.01	4.84
191029A011	1551.6	38.48	14.16	3.56	1.61	0.42	8.31	1278.83	17.56	32.75	0.36	202.40	0.77
191029A012	1551.6	18.62	14.45	3.70	1.09	0.53	5.62	1690.11	15.33	14.68	0.11	198.08	0.47
191029A013	1551.6	25.57	9.56	3.57	2.07	0.33	7.68	1397.07	20.90	40.96	0.40	179.90	0.70
191029A017	1551.6	2.23	1.14	4.32	2.04	0.55	11.17	3660.12	7.80	32.56	0.39	19.17	0.76
191029A018	1551.6	0.53	0.17	5.35	3.30	0.24	12.10	685.39	15.49	66.56	1.38	0.54	1.11
191029A019	1551.6	0.16	0.15	3.02	2.77	0.39	10.82	643.24	18.31	47.91	0.61	0.15	1.00
191029A020	1551.6	118.38	53.40	243.53	103.20	8.49	548.69	3689.77	271.43	656.89	33.16	256.79	36.40
191029A021	1551.6	97.52	39.37	205.65	98.92	6.80	525.59	3703.06	255.12	579.71	42.83	203.83	35.07
191029A022	1551.6	128.71	33.71	338.28	180.30	15.45	900.06	3510.63	248.69	1146.23	50.64	61.89	63.45
191029A023	1551.6	161.58	68.92	482.13	187.47	11.22	1007.08	4030.67	224.68	1102.93	79.71	302.35	70.69
191029A024	1551.6	30.53	21.04	3.10	0.61	0.00	2.59	615.03	20.76	14.05	0.32	19.53	0.17
191029A025	1551.6	0.38	0.20	0.57	0.18	0.24	0.80	310.18	20.12	7.40	0.12	0.08	0.04
191029A026	1551.6	0.66	0.58	0.91	0.29	0.07	1.28	274.17	13.50	7.43	0.18	0.22	0.12
191029A030	1551.6	4.80	10.13	30.88	1.48	0.10	6.27	2425.15	5.81	29.00	0.66	4.40	0.45
191029A031	1551.6	1.92	2.71	4.86	1.05	0.20	5.23	5438.49	8.36	3.04	0.82	3.95	0.44
191029A032	1551.6	12.65	8.44	13.92	7.56	0.32	40.06	3251.29	15.54	47.95	3.35	21.48	2.82
191029A033	1551.6	7.06	8.21	19.93	7.39	0.63	33.70	1055.50	36.58	88.04	2.45	7.62	2.40
191029A034	1551.6	4.87	5.65	10.56	0.88	2.51	7.40	60.75	3.73	55.11	2.14	126.91	0.37
191029A035	1551.6	413.53	117.18	35.29	0.24	2.37	1.32	28.16	2.81	17.51	0.33	616.82	0.11
191029A036	1551.6	42549.82	18333.78	1275.13	241.18	115.80	1109.27	1588.54	57.04	1149.56	68.22	59881.62	134.64
191029A037	1551.6	2209.09	1267.74	118.65	3.88	15.80	18.41	1504.38	20.59	86.52	4.46	4556.78	1.57
191029A038	1551.6	11.84	11.92	2.81	0.39	2.28	2.84	38.83	3.04	50.35	1.90	114.45	0.14
191029A039	1551.6	11.62	12.02	4.35	0.69	2.22	5.92	64.20	3.88	82.59	7.01	110.19	0.29
191029A043	1551.6	3.54	6.67	4.55	0.30	2.26	1.21	23.50	2.43	52.47	1.91	69.21	0.12
191029A044	1551.6	5.05	8.35	7.36	0.63	2.31	4.76	47.51	3.43	63.05	1.95	112.34	0.29
191029A045	1551.6	7.03	7.16	4.92	0.74	2.13	5.76	57.95	3.51	55.59	3.26	106.55	0.29
191029A046	1551.6	555.04	369.17	233.81	1.53	2.24	4.87	84.48	2.55	34.04	1.45	394.87	0.58
191029A047	1551.6	547.14	294.02	28.34	0.55	2.09	1.64	24.09	1.09	12.97	0.56	613.16	0.18
191029A048	1551.6	405.49	116.95	27.11	0.47	1.93	2.29	374.60	1.55	11.31	0.25	372.92	0.20
191029A049	1551.6	857.45	377.56	44.17	1.74	5.95	7.62	1956.38	7.43	52.61	1.34	849.90	0.66
191029A050	1551.6	1.09	3.07	8.68	2.24	0.17	11.16	1329.30	13.66	50.80	0.84	0.55	0.76
191029A051	1551.6	3.39	2.45	12.63	5.42	0.24	31.85	1053.95	17.96	84.52	2.84	1.55	2.33
191029A052	1551.6-2	32.30	7.14	8.98	6.42	1.53	33.57	950.49	18.86	146.53	2.12	0.99	1.64
191029A056	1551.6-2	3.40	1.42	4.04	1.59	0.38	10.85	558.07	26.15	17.93	0.19	1.43	0.24
191029A057	1551.6-2	1.07	1.44	4.03	1.21	0.10	6.62	519.31	27.48	13.23	0.39	1.03	0.44
191029A058	1551.6-2	0.32	0.36	22.38	5.46	0.40	16.25	668.04	22.03	110.09	3.90	0.80	1.19
191029A059	1551.6-2	2.79	0.84	5.80	0.51	2.40	2.72	34.83	7.63	37.36	1.03	140.22	0.28
191029B012	1574.4	0.53	0.47	0.94	0.16	0.27	0.24	358.95	6.96	3.99	0.06	0.00	0.02
191029B013	1574.4	1591.40	485.61	38.26	15.10	11.49	76.61	1408.27	41.42	30.46	3.25	24.88	3.16
191029B017	1574.4	4.93	3.84	9.95	1.85	0.34	9.05	1342.07	34.14	3.65	0.46	0.34	0.42
191029B018	1574.4	16.83	12.33	11.39	2.24	0.65	10.21	1351.44	40.11	5.50	1.72	2.91	0.49
191029B019	1574.4	4.001099175	4.513018453	8.266320784	2.214818602	0.199673927	9.150365394	1319.037119	39.91208974	5.274802005	0.505822829	0.667737799	0.477195853

Contents (ESIS)		Ba	Hf	Ta	W	Tl	Pb	Th	U	La	Ce	Pr	
Point NO.	Sample NO.	ppm	ppm	ppm	ppm	ppm	ppm	ppm	ppm	ppm	ppm	ppm	
191029A004	1551.6	509.78	0.83	0.11	0.93	0.08	2.04	2.79	4.38	24.54	40.94	4.36	
191029A005	1551.6	330.14	0.43	0.05	0.53	0.02	0.35	1.18	1.26	14.79	23.62	2.31	
191029A006	1551.6	226.48	0.51	0.04	0.24	0.04	7.27	3.54	7.50	8.91	21.01	2.98	
191029A007	1551.6	346.00	0.49	0.02	0.47	0.00	0.11	0.42	1.03	18.22	29.76	3.00	
191029A008	1551.6	483.96	1.15	0.08	0.60	0.03	7.90	11.91	3.50	33.40	63.58	6.79	
191029A009	1551.6	4988.56	18.46	2.72	11.62	1.37	202.17	78.50	33.84	242.69	446.08	45.98	
191029A010	1551.6	4429.60	6.09	0.12	6.71	2.28	69.98	13.82	10.10	136.97	219.32	22.85	
191029A011	1551.6	380.26	0.66	0.04	0.32	0.70	22.56	9.38	3.78	17.30	33.19	3.44	
191029A012	1551.6	416.77	0.32	0.01	0.19	0.69	13.68	7.22	2.83	12.35	23.37	2.54	
191029A013	1551.6	501.51	0.64	0.04	0.41	0.36	14.62	13.96	4.41	28.50	56.54	5.94	
191029A017	1551.6	766.14	0.86	0.04	0.30	0.06	4.05	1.23	1.44	6.80	11.29	1.20	
191029A018	1551.6	419.19	1.27	0.14	0.51	0.03	0.62	6.62	2.42	17.44	32.40	3.51	
191029A019	1551.6	353.20	0.91	0.07	0.30	0.02	0.47	5.71	2.89	21.30	40.44	4.36	
191029A020	1551.6	2689.05	16.33	2.58	13.39	2.10	24.28	34.54	85.28	215.39	438.57	50.98	
191029A021	1551.6	2677.72	14.08	3.23	16.12	2.55	19.88	41.16	81.14	208.28	409.20	46.88	
191029A022	1551.6	3322.60	24.97	3.91	18.58	4.16	21.46	63.96	90.52	225.81	413.82	45.98	
191029A023	1551.6	3370.51	26.70	5.98	27.73	4.87	40.30	70.46	89.30	217.49	366.64	41.78	
191029A024	1551.6	482.57	0.28	0.02	0.40	0.29	43.19	1.53	4.07	3.85	7.72	0.96	
191029A025	1551.6	157.94	0.12	0.01	0.08	0.00	0.08	1.70	4.23	2.35	6.20	0.97	
191029A026	1551.6	160.93	0.14	0.01	0.13	0.00	0.25	1.18	3.12	2.00	5.21	0.78	
191029A030	1551.6	144.47	0.52	0.05	0.27	0.06	4.64	0.82	1.13	3.50	5.57	0.50	
191029A031	1551.6	148.50	0.06	0.04	0.18	0.02	0.41	1.10	0.68	8.84	15.90	1.53	
191029A032	1551.6	551.50	1.28	0.26	1.26	0.17	4.58	2.88	4.18	14.02	24.73	2.55	
191029A033	1551.6	481.75	1.24	0.19	1.25	0.17	4.97	2.40	6.70	41.16	71.85	7.30	
191029A034	1551.6	52.82	1.65	0.20	0.79	2.33	15.07	3.72	1.21	2.87	5.74	0.62	
191029A035	1551.6	23.40	0.60	0.03	0.10	35.52	165.27	2.29	0.48	2.78	6.18	0.84	
191029A036	1551.6	2225.62	57.50	7.18	32.79	2239.33	39463.14	21.08	22.92	30.53	51.78	4.91	
191029A037	1551.6	1009.67	3.10	0.41	1.38	188.13	2341.69	11.28	4.85	12.09	26.77	2.93	
191029A038	1551.6	30.62	1.48	0.14	0.59	1.64	49.31	3.92	1.09	0.99	1.67	0.21	
191029A039	1551.6	49.78	1.96	0.42	2.34	2.13	34.91	4.37	1.71	1.62	3.06	0.32	
191029A043	1551.6	20.76	1.58	0.16	0.59	1.56	10.32	3.06	0.76	1.07	1.97	0.24	
191029A044	1551.6	35.35	1.85	0.17	0.59	2.49	12.29	3.79	1.01	1.53	3.09	0.35	
191029A045	1551.6	57.08	1.64	0.16	0.72	1.66	27.62	4.41	1.12	2.08	3.83	0.43	
191029A046	1551.6	77.42	0.86	0.10	0.34	49.19	435.81	2.15	0.49	2.94	5.85	0.65	
191029A047	1551.6	26.15	0.39	0.05	0.23	23.82	1072.59	1.73	0.23	0.75	1.51	0.19	
191029A048	1551.6	371.51	0.19	0.01	0.11	21.17	1347.95	0.78	0.27	1.76	3.35	0.38	
191029A049	1551.6	537.54	0.89	0.08	1.10	35.56	4332.49	2.94	1.27	10.75	20.36	2.16	
191029A050	1551.6	384.69	0.64	0.06	0.82	0.05	0.90	2.28	2.11	14.01	22.92	2.36	
191029A051	1551.6	520.67	1.15	0.18	1.40	0.13	1.18	3.85	4.35	25.08	38.94	3.85	
191029A052	1551.6-2	729.80	3.05	0.15	1.44	0.11	7.15	5.70	3.70	16.50	27.46	2.85	
191029A056	1551.6-2	530.68	0.16	0.01	0.37	0.04	0.81	2.61	4.46	10.04	19.13	2.22	
191029A057	1551.6-2	357.89	0.30	0.03	0.29	0.02	0.37	2.51	4.85	8.42	15.23	1.89	
191029A058	1551.6-2	478.17	2.91	0.24	0.79	0.02	1.82	8.03	3.15	23.55	44.39	4.68	
191029A059	1551.6-2	41.08	1.29	0.15	0.89	1.88	28.73	2.79	1.11	7.05	13.61	1.44	
191029B012	1574.4	993.26	2.02	4.98	0.58	3.25	0.75	0.21	0.90	0.16	0.83	0.19	
191029B013	1574.4	1401.87	24.44	56.64	7.04	34.51	8.58	1.62	6.44	1.39	6.96	1.57	
191029B017	1574.4	115.28	15.71	37.66	4.49	21.71	4.96	1.05	6.37	0.88	5.35	1.11	
191029B018	1574.4	112.03	18.26	43.75	5.48	27.34	6.63	1.26	6.69	0.99	6.56	1.53	
191029B019	1574.4	104.3035504	20.02294836	46.55645951	6.316230251	29.19869965	6.495348197	1.404005987	7.438995138	1.037236591	6.590222254	1.458832083	

Contents (ESIS)		Nd	Sm	Eu	Gd	Tb	Dy	Ho	Er	Tm	Yb	Lu	
Point NO.	Sample NO.	ppm	ppm	ppm	ppm	ppm	ppm	ppm	ppm	ppm	ppm	ppm	
191029A004	1551.6	17.57	3.63	0.81	3.52	0.55	3.91	0.83	2.36	0.38	2.86	0.45	
191029A005	1551.6	8.69	1.63	0.44	1.56	0.26	1.81	0.39	1.16	0.18	1.35	0.22	
191029A006	1551.6	15.98	4.27	1.15	5.49	0.76	5.18	1.15	3.40	0.48	3.57	0.58	
191029A007	1551.6	12.02	2.07	0.39	1.88	0.25	1.61	0.38	1.09	0.16	1.25	0.22	
191029A008	1551.6	26.09	4.73	0.81	3.61	0.60	3.88	0.77	2.40	0.38	2.88	0.44	
191029A009	1551.6	179.42	32.92	6.06	28.05	4.19	27.97	5.95	18.52	2.98	22.08	3.94	
191029A010	1551.6	94.93	16.71	3.83	16.39	2.54	13.29	3.45	10.20	1.32	8.96	1.82	
191029A011	1551.6	13.62	2.55	0.50	2.33	0.34	2.20	0.49	1.68	0.25	1.85	0.28	
191029A012	1551.6	10.13	1.98	0.34	1.71	0.26	1.69	0.38	1.27	0.18	1.48	0.26	
191029A013	1551.6	22.66	4.09	0.77	3.69	0.48	3.28	0.76	2.07	0.32	2.47	0.38	
191029A017	1551.6	4.68	0.87	0.17	0.83	0.09	0.69	0.20	0.56	0.09	0.74	0.16	
191029A018	1551.6	13.80	2.16	0.44	1.98	0.34	1.98	0.44	1.36	0.21	1.69	0.30	
191029A019	1551.6	16.72	2.88	0.52	2.69	0.39	2.68	0.57	1.72	0.31	2.10	0.38	
191029A020	1551.6	218.07	44.41	10.01	43.97	6.58	39.40	8.21	22.46	3.25	19.85	3.22	
191029A021	1551.6	195.33	41.08	8.96	38.34	6.30	37.81	7.65	22.88	3.02	19.55	2.98	
191029A022	1551.6	180.74	41.08	8.44	36.37	5.38	35.57	7.90	22.00	3.39	25.12	3.96	
191029A023	1551.6	164.17	35.03	6.92	33.75	5.01	32.00	6.69	19.13	3.24	23.11	3.69	
191029A024	1551.6	5.16	1.59	0.44	2.13	0.27	2.21	0.44	1.68	0.24	1.70	0.29	
191029A025	1551.6	5.34	1.52	0.36	1.84	0.29	2.04	0.51	1.49	0.26	1.75	0.34	
191029A026	1551.6	4.20	1.11	0.28	1.29	0.18	1.30	0.33	1.08	0.17	1.31	0.26	
191029A030	1551.6	1.87	0.45	0.11	0.57	0.09	0.70	0.16	0.59	0.10	0.65	0.13	
191029A031	1551.6	5.64	0.87	0.14	0.70	0.11	0.88	0.23	0.69	0.11	0.81	0.17	
191029A032	1551.6	9.78	1.95	0.46	1.84	0.28	1.86	0.42	1.40	0.21	1.70	0.27	
191029A033	1551.6	28.96	6.07	1.08	5.17	0.78	5.36	1.20	3.42	0.60	4.47	0.70	
191029A034	1551.6	2.31	0.47	0.07	0.34	0.08	0.52	0.14	0.48	0.08	0.68	0.10	
191029A035	1551.6	3.70	0.84	0.18	0.68	0.09	0.50	0.10	0.27	0.04	0.30	0.04	
191029A036	1551.6	21.23	6.05	0.68	4.05	1.03	5.61	1.46	6.39	0.99	8.18	1.57	
191029A037	1551.6	12.57	2.65	0.47	1.96	0.37	2.52	0.61	1.91	0.33	2.35	0.29	
191029A038	1551.6	0.89	0.26	0.04	0.23	0.05	0.40	0.10	0.33	0.06	0.48	0.08	
191029A039	1551.6	1.14	0.34	0.06	0.32	0.07	0.58	0.14	0.52	0.08	0.79	0.10	
191029A043	1551.6	0.91	0.23	0.05	0.22	0.04	0.35	0.09	0.27	0.04	0.41	0.07	
191029A044	1551.6	1.45	0.27	0.06	0.35	0.08	0.67	0.10	0.40	0.06	0.53	0.08	
191029A045	1551.6	1.67	0.43	0.06	0.37	0.08	0.54	0.16	0.35	0.07	0.65	0.09	
191029A046	1551.6	2.76	0.48	0.10	0.38	0.08	0.45	0.10	0.24	0.04	0.34	0.04	
191029A047	1551.6	0.93	0.15	0.03	0.14	0.02	0.15	0.03	0.11	0.02	0.07	0.01	
191029A048	1551.6	1.57	0.23	0.06	0.23	0.03	0.22	0.05	0.14	0.03	0.16	0.03	
191029A049	1551.6	8.13	1.31	0.28	1.11	0.18	1.08	0.27	0.85	0.13	0.73	0.15	
191029A050	1551.6	9.15	1.94	0.48	1.78	0.26	1.80	0.40	1.20	0.17	1.34	0.20	
191029A051	1551.6	15.84	2.89	0.66	2.87	0.40	2.72	0.59	1.76	0.27	1.73	0.30	
191029A052	1551.6-2	11.95	2.71	0.71	2.62	0.27	2.02	0.43	1.35	0.24	1.73	0.31	
191029A056	1551.6-2	10.47	2.16	0.57	2.66	0.43	2.70	0.62	2.02	0.28	2.29	0.42	
191029A057	1551.6-2	8.84	2.15	0.53	2.55	0.40	2.54	0.65	2.17	0.35	2.46	0.44	
191029A058	1551.6-2	18.34	3.36	0.65	3.08	0.41	2.95	0.56	1.90	0.32	2.35	0.40	
191029A059	1551.6-2	5.82	1.16	0.19	1.08	0.17	1.15	0.29	0.96	0.17	1.38	0.21	
191029B012	1574.4	0.55	0.08	0.75	0.12	0.06	0.00	0.04	0.01	0.54	1.27	0.97	
191029B013	1574.4	4.40	0.44	3.45	0.56	0.72	0.25	1.05	1.28	2427.98	23.31	3.41	
191029B017	1574.4	3.30	0.50	3.36	0.52	0.11	0.05	0.15	0.07	4.30	7.60	6.58	
191029B018	1574.4	4.23	0.59	4.23	0.66	0.15	0.20	0.53	0.07	38.77	13.04	8.55	
191029B019	1574.4	3.776425533	0.521954776	3.751718662	0.5451307	0.127297297	0.033166793	0.19651884	0.075238107	1.627000278	11.16199606	11.15435165

From the standard REE distribution pattern of chondrite in the sample (Fig. 6b,d,f,h,j), it can be seen that the changing characteristics of the curve are basically the same, showing that the fractionation is obvious between the LREE and HREE; thus, the LREE is enriched and the HREE is relatively depleted. ① In sample 1551.6, the REE curve at the position of blue line No. 1 is relatively flat, the Eu content at some points shows a positive anomaly, and the Eu content at some points is normal, indicating that the formation temperature of the channel changes. The REE curve at the position of blue line No. 2 is relatively flat, and all points of the Eu content show a weak negative anomaly. The REE curve of the pyrite layer in the No. 3 blue line is relatively flat. Some points of the Eu content show positive anomalies, and some points of the Eu content are normal, indicating that the formation temperature of the channel changes; in addition, the mineral formation temperature near the hydrothermal chimney is higher. However, away from the hydrothermal chimney, the mineral formation temperatures are low. ② In sample 1574.4, the curve of LREE is steeper, the curve of HREE is relatively flat, and all points of the Eu content show a weak negative anomaly. ③ In sample 1551.6–2-1, the curve of LREE is steep, the curve of HREE is relatively flat, and all points of the Eu content show a weak negative anomaly.

### Abnormalities of in situ S isotopes of pyrite

Traditional S, Pb isotope analysis mostly uses single mineral powder samples (bulk analysis). It is difficult to guarantee the purity of single mineral particles, and it is difficult to separate the single mineral samples formed under different genesis. Simultaneously, it is difficult to finely distinguish the ring bands formed under different conditions for the sulphides of the developmental ring structure, so that the test results are often indicating multi-sources. Mixed values result in multiple solutions. NanoSIMS is capable of in situ micro-analysis of micron-sized pyrite. It can accurately analyse single mineral particles without destroying the particle structure and has high spatial resolution, which replaces the shortcomings of traditional analytical methods. There are four main sources of sulphur on the earth: (1) mantle sulphur (magma sulphur), δ^34^S value is close to 0, usually 0 ± 3‰; (2) seawater sulphur, usually characterized by a large positive value; (3) sedimentary sulphur, in which the sulphur isotopic composition varies greatly (− 40 ‰ ~ 50 ‰); and (4) mixed sulphur, involving the mixing of two or more different sources of sulphur, in which the level of the sulphur isotope is usually determined by the sulphur isotope composition and mixing^11^. A fifth source of sulphur includes the sulphur reduction of hydrogen sulphide and sulphur compounds by bacteria, which is generally less than -5‰. Petrological observations show that the Chang 7 high-quality source rocks have the characteristics of anomalous pyrite enrichment, with different morphologies, including octahedron self-crystals, irregular microcrystalline aggregates with framboidal filling, and large framboidal morphologies. This shows that during the Chang 7 sedimentary period, the sulphate supply required for pyrite formation was sufficient. The sulphur isotope data of pyrite in the source rocks of the Zh22 well are shown in Table 3. The δ^33^Sv-CDT values of the 33 points ranged from 1.50 to 5.30, with an average of 4.883. The δ^34^Sv-CDT value ranged from 7.89 to 10.88, with an average of 9.591. The sulphur isotope characteristics of pyrite and raspberry pyrite in the Chang 7 high-quality source rock, the consistency of the sulphur isotopes of the pyrite in the longitudinal direction of the Chang 7 high-quality hydrocarbon source, and the similar sulphur isotope composition characteristics of tuff, aggregate pyrite and raspberry pyrite, reflect that sulphur compounds in hydrothermal activity at the bottom of the lake may be one of the important sources of sulphur.

**Table 3:** S isotope data. We completed a total of 19 points of in situ S isotope experiments on sample 1551.6. The experimental position corresponds to blue line 3 in Fig. 5a.

No	Sample name	33/32S	SE	34/32S	SE	Total Beam (V)	SE	δ^33^S_ref_	δ^33^S_v-CDT_	δ^34^S_ref_	δ^34^S_v-CDT_	2SE
1	Z22-1551.6 py	0.0083788	1.25E−06	0.0493574	3.55E−06	7.44	0.07	3.60	4.48	7.67	9.38	0.14
2	Z22-1551.6 py	0.0083707	1.39E−06	0.049402	4.21E−06	6.63	0.10	3.84	4.72	8.37	10.09	0.17
3	Z22-1551.6 py	0.0083677	1.55E−06	0.049374	4.23E−06	5.03	0.14	3.48	4.36	7.80	9.51	0.17
4	Z22-1551.6 py	0.0083547	2.22E−06	0.0492766	0.0000048	5.26	0.09	3.90	4.78	5.80	7.51	0.19
5	Z22-1551.6 py	0.0083474	1.85E−06	0.0492661	5.44E−06	3.99	0.10	3.02	3.90	5.58	7.29	0.22
6	Z22-1551.6 py	0.0083561	1.73E−06	0.0493859	4.49E−06	4.65	0.07	4.34	5.22	7.95	9.66	0.18
7	Z22-1551.6 py	0.0083473	1.97E−06	0.0492777	4.27E−06	4.54	0.13	3.29	4.17	5.74	7.45	0.17
8	Z22-1551.6 py	0.0083563	1.28E−06	0.0494101	3.69E−06	7.32	0.10	3.82	4.70	8.24	9.95	0.15
9	Z22-1551.6 py	0.0083629	1.24E−06	0.0493875	0.0000034	7.10	0.09	4.62	5.50	7.77	9.49	0.14
10	Z22-1551.6 py	0.008358	1.51E−06	0.0493663	3.36E−06	5.85	0.09	3.65	4.53	7.18	8.89	0.14
11	Z22-1551.6 py	0.0083587	1.25E−06	0.0493892	3.34E−06	7.40	0.08	3.73	4.61	7.64	9.36	0.14
12	Z22-1551.6 py	0.0083598	1.11E−06	0.049388	3.98E−06	7.15	0.08	4.28	5.15	7.61	9.32	0.16
13	Z22-1551.6 py	0.0083446	1.41E−06	0.0492965	4.21E−06	6.08	0.21	2.45	3.32	5.74	7.45	0.17
14	Z22-1551.6 py	0.008356	0.0000011	0.0493938	3.63E−06	7.73	0.07	4.42	5.30	7.72	9.43	0.15
15	Z22-1551.6 py	0.0083476	1.41E−06	0.049322	0.0000037	6.44	0.12	3.42	4.30	6.25	7.96	0.15
16	Z22-1551.6 py	0.0083489	1.03E−06	0.0493467	0.0000037	7.77	0.09	4.02	4.90	6.77	8.48	0.15
17	Z22-1551.6 py	0.008343	1.31E−06	0.0492919	3.33E−06	7.11	0.10	3.31	4.19	5.65	7.36	0.14
18	Z22-1551.6 py	0.0083508	1.29E−06	0.0494014	0.0000042	7.47	0.09	4.33	5.21	7.87	9.58	0.17
19	Z22-1551.6 py	0.0083453	1.17E−06	0.0492936	3.44E−06	7.51	0.09	3.67	4.55	5.67	7.38	0.14
20	Z22-1551.6 py	0.0083484	1.06E−06	0.049383	3.51E−06	6.69	0.13	3.81	4.69	7.52	9.23	0.14
21	Z22-1551.6 py	0.0083404	1.52E−06	0.049294	3.55E−06	6.06	0.13	2.85	3.73	5.70	7.41	0.14
22	Z22-1551.6 py	0.0083536	0.0000016	0.049387	5.12E−06	7.37	0.10	4.35	5.23	7.69	9.40	0.21
23	Z22-1551.6 py	0.0083416	4.81E−06	0.0492965	8.51E−06	1.78	0.11	2.91	3.79	5.84	7.55	0.35
24	Z22-1551.6 py	0.0083433	1.35E−06	0.0493038	3.87E−06	5.80	0.16	3.18	4.06	5.95	7.66	0.16
25	Z22-1551.6 py	0.0083448	1.15E−06	0.0493251	3.75E−06	7.39	0.11	3.36	4.24	6.39	8.10	0.15
26	Z22-1551.6 py	0.0083478	1.35E−06	0.0493463	0.0000038	5.84	0.11	4.02	4.89	6.83	8.54	0.15
27	Z22-1551.6 py	0.0083401	1.29E−06	0.0493239	4.46E−06	5.46	0.19	3.09	3.96	6.37	8.08	0.18
28	Z22-1551.6 py	0.0083416	3.23E−06	0.049277	6.68E−06	2.86	0.13	3.31	4.19	5.49	7.20	0.27
29	Z22-1551.6 py	0.0083454	1.46E−06	0.04933	0.0000037	6.30	0.04	3.77	4.64	6.57	8.28	0.15
30	Z22-1551.6 py	0.0083445	1.38E−06	0.0493391	3.28E−06	6.87	0.07	3.73	4.61	6.78	8.49	0.13
31	Z22-1551.6 py	0.0083434	1.39E−06	0.0493948	3.55E−06	5.97	0.10	3.59	4.47	7.91	9.63	0.14
32	Z22-1551.6 py	0.0083452	1.49E−06	0.0494052	3.97E−06	5.74	0.12	4.02	4.90	8.16	9.87	0.16
33	Z22-1551.6 py	0.0083455	1.05E−06	0.0494037	3.69E−06	6.79	0.07	4.06	4.94	8.13	9.84	0.15

## Discussion

### Identification of the cause of hydrothermal chimneys

**Figure 7:**
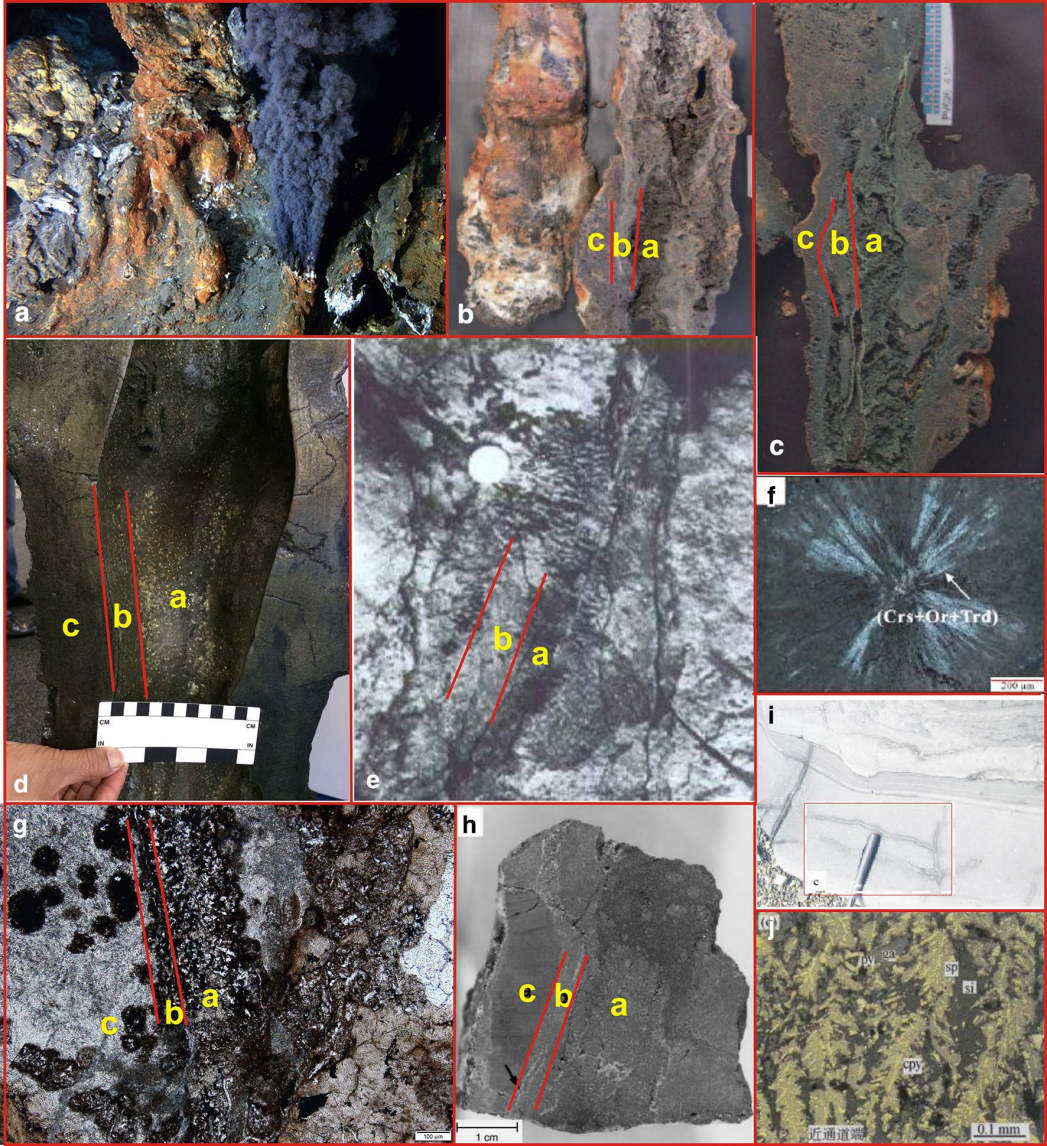
Comparison with previous research results. (**a**) Hydrothermal vent field near the Mid-Atlantic Ridge at 30° N^5^. (**b**) The outline of the black chimney of the Brothers submarine volcano^28^. (**c**) Black chimney section of the Brothers submarine volcano^28^. (**d**) The black chimney channel from the South African Geological Museum. Layer a in the figure is the outer wall of the channel, and layer b is the inner wall of the channel(Photo was taken by Liu Yiqun). (**e**) Photograph of hydrothermal sedimentary dolomite in a natural outcrop in the Sardinian mining area of Italy^22^, similar to the shape of the hydrothermal chimney in Fig. 3b. (**f**) The radial structure of the North Island hydrothermal zone, New Zealand^25^ is similar to the radial structure of Fig. 3g. (**g**) Channel of the hydrothermal chimney in this study; layer a in the figure is the outer wall of the channel, and layer b is the inner wall of the channel. (**h**) TAG hydrothermal mound, Mid-Atlantic Ridge, 26° N^23^, similar to the channel structure of the study area in Fig. 3b. (**i**) Drainage structure. (**j**) Black chimney in North China^12^.

Predecessors have carried out detailed studies on hundreds of active hydrothermal chimneys and obtained preliminary important information on the geometry, geological characteristics, internal structure, and mineral composition of the chimneys, which can also be used as an effective method to distinguish hydrothermal chimneys in the geological record^12^. Through comparative studies, the mineral composition and structure of the hydrothermal chimneys in this paper are very similar to those reported in previous studies on modern continental and oceanic hydrothermal chimneys. ① The channels of the hydrothermal chimneys found in the study area are similar in appearance to the drainage structure of the sedimentary rocks, but the two are essentially different. The drainage structure is mostly found in fine sandstone and black mudstone. The planes form irregular polygons, similar to the cracks on a turtle shell. The cracks formed by the drainage structure are limited to the layer and the irregular mesh structure. The sandstone in the lower part of the vertical section is liquefied, and the upper mudstone is located within fine veins and finally penetrates to the top surface of the mudstone to complete the discharge (Fig. 7i). The jet channel in Fig. 2e is filled with calcite and dolomite crystals; a large number of radial and saddle-like dolomite, euhedral pyrite and elongated anhydrite crystals have developed around this area; the top consists of a flower-like calcite structure, and there are cryptocrystalline structures with stacked cone-shaped, leaf-like and harbor-like shapes. These structures are related to hot air waves at the bottom of the lake. ② The most significant structural feature of modern hydrothermal chimneys is that both the section perpendicular to the chimney and the section parallel to the chimney show zoning. The former is manifested as the zoning of the channel, the inner wall and the outer wall; the latter is often manifested as the formation of a dendritic shape in the upper part of the chimney, retaining the spout structure. The phenomenon of chimney merging or branching is very common, and multiple chimneys frequently cluster to form complex shapes. These include the hydrothermal chimney from the Geological Museum in South Africa (Fig. 7d), the TAG hydrothermal mound (Fig. 7h), and the Brothers submarine volcano (Fig. 7b,c). The hydrothermal chimney in this study (Fig. 7g) is similar in structure to hydrothermal chimneys from modern oceanic environments, so the research method of modern oceanic hydrothermal chimneys is applied(Fig. 7a). ③ On the horizontal section, modern hydrothermal chimneys often show concentric circles or complex mineral zoning^13^. The outer wall of the chimney is composed of anhydrite, dolomite, calcite and pyrite. The inner wall is often composed of coarse-grained sphalerite, pyrite, and anhydrite. The plate-like minerals grow vertically on the inner wall and are arranged in a concentric shape and radiate outward. The rock mineral combination of the black chimney in North China is siliceous rock and pyrite + anhydrite (Fig. 7j). Hydrothermal dolomite developed in the mining area of Sardinia, Italy (Fig. 7e); the radial structure of dolomite is developed in the hydrothermal area of North Island, New Zealand (Fig. 7f).

**Figure 8:**
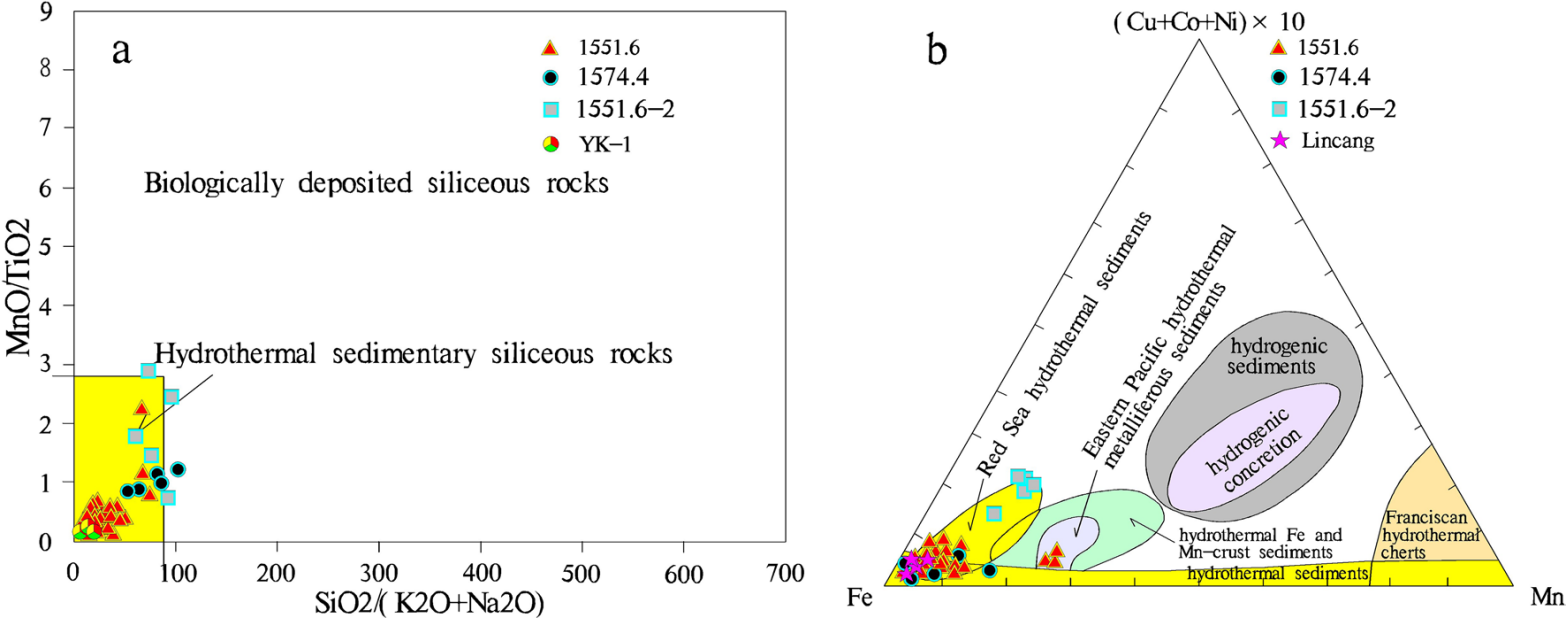
Discrimination diagrams for sedimentary rocks from the Zh22 well in the Ordos Basin (All samples are hydrothermal dolomite). (**a**) SiO_2_/(K_2_O + Na_2_O)-MnO/TiO_2_^15^. The data of 1551.6, 1574.4 and 1551.6–2 are from this study, and the data of YK-1 is from Reference^7^. (**b**) Fe–Mn-(Cu + Co + Ni) × 10^14^. The data of 1551.6, 1574.4 and 1551.6-2 are from this study, and the data of YK-1 is from Reference^16^.

On the basis of research on the structure, petrology, mineralogy, and geochemical characteristics of the hydrothermal chimney and the source of the S component in breccia pyrite, we have identified the mechanism of the hydrothermal chimney samples. Generally, hydrothermal activity related to chimneys generates abundant element sources for minerals. After the deposition of hydrothermal substances, the abundances of elements in the sediments show anomalies, and they spread around the nozzle^4^. The rocks formed are defined as hydrothermal sedimentary rocks, which are the product of precipitation after the hydrothermal fluid is ejected from the bottom of the sea/lake and interacts with the sea/lake water. Previous researchers have established a systematic and multi-index coexistence judgment criterion for hydrothermal deposition through a large amount of research on marine hydrothermal deposition, and a geochemical discrimination diagram is one of the methods used. Here, the Fe–Mn-(Cu + Co + Ni) × 10 triangle^14^ (Fig. 8b) and the SiO_2_/(K_2_O + Na_2_O)-MnO/TiO_2_ diagram^15^ (Fig. 8a) are successfully applied to the identification of terrestrial hydrothermal deposition^8,16^. The study area in this paper is characterized by typical lacustrine hydrothermal deposition. Therefore, this paper refers to the geochemical discriminant diagrams for hydrothermal sedimentary rocks to support the formation of hydrothermal chimneys. In the SiO_2_/(K_2_O + Na_2_O)-MnO/TiO_2_ discrimination diagram of the sediment (Fig. 8a), all projection points of the 1551.6 sample plot in the hydrothermal deposition zone; most of the projection points of the 1574.4 sample and the 1551.6–2 sample plot in the hydrothermal deposition zone, and a small number of the projection point plot close to the hydrothermal-rich deposition zone. In the Fe–Mn-(Cu + Co + Ni) × 10 discrimination diagram (Fig. 8b), some projection points of the 1551.6 sample plot in the hydrothermal deposition area, some projection points plot in the hydrothermal deposition area of the Red Sea, and there are 3 projection points that plot in the eastern Pacific Ocean hydrothermal sedimentation area. Some projection points of the 1574.4 sample plot in the hydrothermal deposition area, and some projection points plot in the hydrothermal deposition area of the Red Sea; some projection points of the 1551.6–2 sample plot in the hydrothermal deposition area of the Red Sea, and other projection points plot close to the hydrothermal-rich deposition zone.

### Formation and growth of hydrothermal chimneys

In the Chang 7 depositional period of the Triassic Yanchang Formation, the Ordos Basin had the characteristics of frequent event sedimentation, strong tectonic activity and activation of basement faults, which created favorable conditions for hydrothermal jet activity^17^. According to the petrological and mineralogical characteristics of hydrothermal chimneys in the study area, we established a hydrothermal chimney formation model for the area (Fig. 2a).

It is believed that the sulfate formation stage and the sulfide and carbonate formation stage are the main stages experienced by the hydrothermal chimney in the study area. ① The chimney walls are initially constructed upward from the sea floor by anhydrite precipitation from seawater. The lake water is in contact with the ejected hydrothermal fluid, the CaSO_4_ in the heated lake water is supersaturated, and anhydrite precipitates out of the seawater, forming a structure with anhydrite as the first main mineral. In the outer skin of the chimney, hydrothermal fluid, which is trapped as microscopic inclusions within anhydrite or leaks out into the exterior by dissolution of anhydrite along cleavage planes, is rapidly cooled and precipitates the fine-grained pyrite assemblage resembling black smoker particulates. Throughout the hot, active life of the chimney, anhydrite continues to build the chimney walls upward and outward at the interface between the chimney and seawater. Metastable pyrrhotite precipitated at the chimney exterior is dissolved and reprecipitated as pyrite when the exterior skin becomes incorporated within the chimney wall by the outward accretion of anhydrite. The anhydrite of the hydrothermal chimney in the study area has a relatively complete crystal form, which is elongated under SEM (Fig. 3e). In addition, the precipitation of anhydrite causes the outer wall of the chimney body to usually have high calcium and strontium contents (Table 2). ② In the formation stage of sulfide and carbonate, due to the formation of the anhydrite wall of the hydrothermal chimney, which prevents direct contact between the hydrothermal fluid and lake water, dolomite, calcite and Cu-Fe-Zn sulfides begin to precipitate in all directions in the chimney and replace the anhydrite. This introduces stage II, which is dominated by carbonate and sulfide precipitation. The chimney at this point is growing upward (by precipitation of anhydrite), inward (by precipitation of carbonate and Cu-Fe sulfides) and outward (by anhydrite accretion and subsequent replacement of anhydrite by carbonates). During this process, deep magmatic-hydrothermal fluid continues to flow up to the bottom of the lake basin. Both the temperature and the pressure decrease sharply to much lower values than the environmental conditions inside the magma channel. Therefore, the magmatic-hydrothermal fluid reacts with the cold water, and the "blasting" effect occurs, causing the first crystallized minerals to break into angular shapes and radial shapes (Fig. 3g), and then accumulate near the hydrothermal chimney. ③ We also found abundant biological fossils around the hydrothermal chimney (Fig. 3i). All fossils have been destroyed and are associated with hydrothermal dolomite. A previous study^18^ explained that the creatures living around the hydrothermal chimney suffered a disaster, died and were quickly buried to form fossils. The distal deposits of the hydrothermal chimney are interbedded dolomite and mudstone (Fig. 3i). The explanation is that at a location far from the hydrothermal chimney, due to the intermittent activity of the hydrothermal fluid, terrigenous clastic deposition and hydrothermal deposition alternately occur^19,20^.

### The environmental indications and petroleum significance of the hydrothermal chimney

Modern black chimney research in the ocean shows that with the waning and end of hydrothermal activity and the effect of seawater dynamics, the black chimney body undergoes continuous erosion and disintegration; in addition, unstable components are dissolved, and eventually, a sulfide deposit is formed. In contrast to the long history of geology, the life span of a black chimney on the ocean floor after its formation is generally short, which is only a part of the sulfide depositional formation process^21^. Therefore, marine and lacustrine hydrothermal chimneys, especially large-scale hydrothermal chimneys, are difficult to preserve during geologic history. The hydrothermal chimneys from the Chang 7 Member of the Triassic Yanchang Formation in the Ordos Basin are mainly on the scale (diameter, height) of a few centimeters to tens of centimeters in size, corresponding to the small-scale chimneys and their top nozzles in modern submarine chimneys. This is also a common feature of hydrothermal activity zones in other parts of the world (Slave Point in Canada, Sardinia in Italy)^22^. From the morphological comparison perspective, the Ordos Basin retains relatively complete paleo-hydrothermal nozzles, which are columnar with bifurcated channels. This arrangement is related to the low-temperature hydrothermal migration rate and the geothermal gradient in the continental rift. From the horizontal bedding developed in the oil shale in the study area, the hydrodynamic conditions at the bottom of the lake were weak. Hydrothermal chimneys are quickly buried and preserved by sediments after the growth and eruption stop. These are the reasons why hydrothermal chimneys of different scales are widely retained in this area with different enrichments.

**Figure 9:**
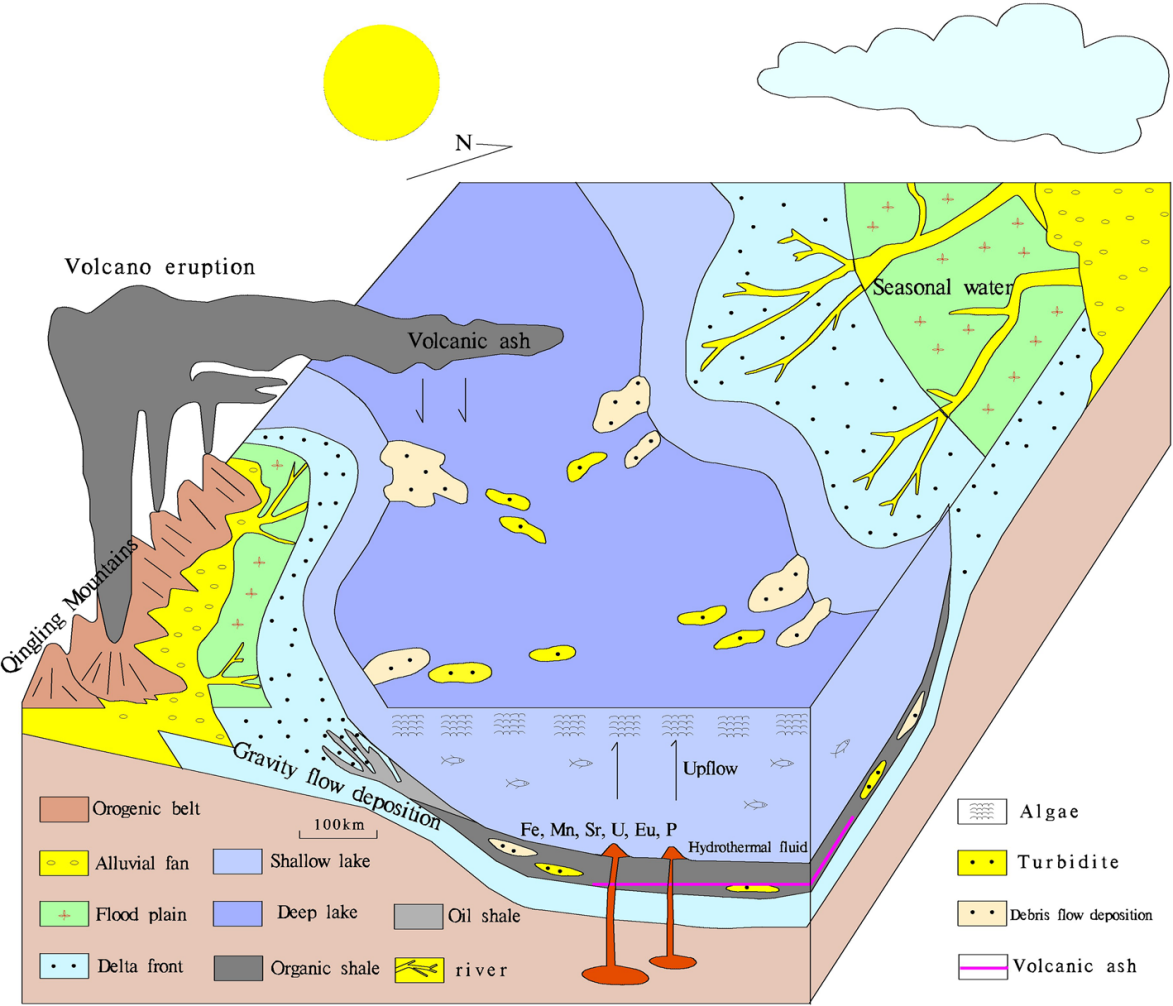
Sedimentary model map of the Late Triassic in the Ordos Basin(Map modified from reference^8^,(The Figure is drawn with Mapgis6.7, version number is 6.7, the map from URL link: https://www.tandfonline.com/doi/full/10.1080/08120099.2019.1612783).

The target layer with the hydrothermal chimneys is located in the Chang 7 section of the Triassic Yanchang Formation in the Ordos Basin and is a high-quality source rock in the study area. The traditional view is that this set of high-quality source rocks is a dark mudstone of the deep lake facies, which is related only to normal lake deposition. Some researchers^17,18^ have suggested that hydrothermal activity promotes the formation of high-quality source rocks. Hydrothermal activity not only provides thermal energy to promote the maturation of hydrocarbons but also causes the convection and circulation of water, bringing bacteria and minerals from the bottom of the lake to the surface water body, thereby promoting algae reproduction and increasing biological productivity. These conditions promote the formation of high-quality source rocks in the Ordos Basin(Fig. 9). In addition, hydrothermally formed petroleum has been discovered in the Gulf of Guaymas Basin^23^, the Escanaba Trough^24^, and the Okinawa Trough^25^. These findings provide a ‘natural laboratory’^26^ that not only sheds light on the petroleum generation process and the behavior of petroleum in high-temperature fluids but also provides a new perspective for the study of hydrocarbon organic matter sources and genesis. The study of hydrothermal chimneys in lacustrine oil shale will help deepen understanding of the development mechanism of source rocks in continental basins and lay a foundation for further research on lacustrine hydrothermal petroleum.

## Conclusion

The petrology, mineral assemblage, structure, geochemistry and isotopic characteristics of the samples show that there are lacustrine hydrothermal chimneys in the Chang 7 member of the Triassic Yanchang Formation in the Ordos Basin.

The formation of the hydrothermal chimneys involved two stages and the formation of mineral zoning across the walls of these structures throughout two major growth phases: a sulfate-dominated stage and a carbonate and sulfide replacement stage. In the second stage, the hydrothermal fluid migrating outward from the center of the chimney dissolved and replaced anhydrite with carbonate and sulfide minerals.

In the Late Triassic, the lake in the Ordos Basin had a great water depth, weak hydrodynamic conditions at the bottom of the lake, and an anoxic environment. The hydrothermal chimneys were quickly buried and preserved by sediments after the growth and eruption stopped. The hydrothermal chimneys are distributed in the black rock series in the center of the lake basin, which is a high-quality source rock. The study of hydrothermal chimneys in high-quality source rocks has provided new context for further understanding the development of petroleum.
